# Prevalence of Australians exposed to potentially cardiotoxic cancer medicines: a population-based cohort study

**DOI:** 10.1016/j.lanwpc.2023.100872

**Published:** 2023-08-02

**Authors:** Benjamin Daniels, Maria Aslam, Marina T. van Leeuwen, Martin Brown, Lee Hunt, Howard Gurney, Monica Tang, Sallie-Anne Pearson, Claire M. Vajdic

**Affiliations:** a Medicines Intelligence Research Program, School of Population Health, UNSW Sydney, Australia; b Cancer Services, Hunter New England Local Health District, Newcastle, Australia; c Equity in Health and Wellbeing Research Program, Hunter Medical Research Institute, Newcastle, Australia; d School of Medicine and Public Health, College of Health, Medicine and Wellbeing, University of Newcastle, Newcastle, Australia; e Centre for Big Data Research in Health, UNSW Sydney, Australia; f Faculty of Medicine and Health Sciences, Macquarie University, Sydney, Australia; g Cancer Voices New South Wales, Sydney, Australia; h Nelune Comprehensive Cancer Centre, Prince of Wales Hospital, Sydney, Australia; i The Kirby Institute, UNSW Sydney, Australia

**Keywords:** Cardio-oncology, Oncology, Cardiovascular disease, Systemic cancer treatment, Chemotherapy

## Abstract

**Background:**

Cardiovascular disease (CVD) and cancer are leading causes of death and people with cancer are at higher risk of developing CVD than the general population. Many cancer medicines have cardiotoxic effects but the size of the population exposed to these potentially cardiotoxic medicines is not known. We aimed to determine the prevalence of exposure to potentially cardiotoxic cancer medicines in Australia.

**Methods:**

We identified potentially cardiotoxic systemic cancer medicines through searching the literature and registered product information documents. We conducted a retrospective cohort study of Australians dispensed potentially cardiotoxic cancer medicines between 2005 and 2021, calculating age-standardised annual prevalence rates of people alive with exposure to a potentially cardiotoxic medicine during or prior to each year of the study period.

**Findings:**

We identified 108,175 people dispensed at least one potentially cardiotoxic cancer medicine; median age, 64 (IQR: 52–74); 57% female. Overall prevalence increased from 49 (95%CI: 48.7–49.3)/10,000 to 232 (95%CI: 231.4–232.6)/10,000 over the study period; 61 (95%CI: 60.5–61.5)/10,000 to 293 (95%CI: 292.1–293.9)/10,000 for females; and 39 (95%CI: 38.6–39.4)/10,000 to 169 (95%CI: 168.3–169.7)/10,000 for males. People alive five years following first exposure increased from 29 (95%CI: 28.8–29.2)/10,000 to 134 (95%CI: 133.6–134.4)/10,000; and from 22 (95%CI: 21.8–22.2)/10,000 to 76 (95%CI: 75.7–76.3)/10,000 for those alive at least 10 years following first exposure. Most people were exposed to only one potentially cardiotoxic medicine, rates of which increased from 39 (95%CI: 38.7–39.3)/10,000 in 2005 to 131 (95%CI: 130.6–131.4)/10,000 in 2021.

**Interpretation:**

The number of people exposed to efficacious yet potentially cardiotoxic cancer medicines in Australia is growing. Our findings can support the development of service planning and create awareness about the magnitude of cancer treatment-related cardiotoxicities.

**Funding:**

10.13039/501100000925NHMRC Centre for Research Excellence in Medicines Intelligence, 10.13039/501100001171Cancer Institute NSW Early Career Fellowship.

**Research in context**

**Evidence before this study**

Current guidelines recommend baseline risk assessment in all cancer patients scheduled to receive potentially cardiotoxic anticancer therapy, with personalised monitoring, risk factor modification, and surveillance following treatment. We searched for studies quantifying exposure to potentially cardiotoxic cancer medicines at a population level. We searched MEDLINE for studies in English from 1996 until March 2023 using terms (cancer) AND (cardiotoxicity) AND (epidemiology) AND (population). None of the 101 studies from this search examined national prevalence estimates for exposure to potentially cardiotoxic cancer therapies. Other keyword combinations of “cancer”, “cancer medicines”, “antineoplastic therapy”, “cancer survivors”, “prevalence”, “rates of exposure”, “population” did not reveal any studies in MEDLINE and Embase addressing this question. Prior research has predominantly focused instead on cardiovascular outcomes for subsets of the cancer population and individual cancer medicines or classes of medicines.

**Added value of this study**

To our knowledge this is the first study to estimate the at-risk population for cardiovascular toxicity associated with treatment with systemic cancer medicines. Our analysis was not restricted by tumour type or class of medicine. We applied a modern definition of cardiotoxicity extending beyond heart failure to include a range of acute and chronic cardiovascular effects as per current guidelines. Our person-level, longitudinal cancer medicines dispensing data is unique amongst national data collections from similar jurisdictions with universal healthcare arrangements, as systemic cancer therapy is captured in Australian dispensing data.

**Implications of all the available evidence**

Our study suggests that the number of people, living with exposure to potentially cardiotoxic medicines is substantial and increasing steadily with time. The complexities of cardiovascular toxicities and increasing role of prevention and surveillance in cardio-oncology necessitate cooperation between health care professionals to ensure care delivery that identifies and mitigates risks. Our findings from the Australian population can inform cancer service planning and guide future research.

## Introduction

Cancer and cardiovascular disease (CVD) are the leading causes of death and major public health concerns with shared risk factors.^1^^,^^2^ People with cancer are also at higher risk of developing CVD than the general population.^2^, ^3^, ^4^, ^5^ As cancer survival outcomes improve, the long-term risk of cardiovascular mortality for some cancer patients eclipses cancer-specific mortality.^4^^,^^6^ There is a growing recognition that health systems must accommodate the broader healthcare needs of survivors.^7^

Many pharmacological cancer treatments are associated with a wide range of cardiovascular complications^8^, ^9^, ^10^, ^11^ that can interfere with completion of cancer therapy and contribute to morbidity and mortality for cancer survivors. Longer-term cardiotoxicity is variable and dynamic, based on a combination of pre-existing risk factors, subsequent cardiac insults, and initiation of treatments for cardiac dysfunction.^12^ Symptoms may not be observed for years, well beyond the time of exposure to specific cancer treatment.

Despite concerns around potential cardiac events and CVD in cancer survivors, the number of people potentially at risk for these outcomes is not known. The aim of our study was to estimate the prevalence of the at-risk population for pharmacological cancer treatment-related CVD in Australia. We used national dispensing data to estimate prevalence rates of people alive in each year from 2005 through 2021 with previous exposure to potentially cardiotoxic cancer medicines in Australia.

## Methods

### Study setting and data

Australia maintains a publicly-funded, universal healthcare system entitling all citizens and permanent residents to subsidised medicines through the Pharmaceutical Benefits Scheme (PBS). All cancer medicines dispensed in the community, private hospitals, and to outpatients in public hospitals are captured in the PBS data collection. The PBS does not subsidise cancer medicines dispensed within public hospitals to public hospital inpatients and, as a result, inpatient treatment is used minimally, with most treatments administered in the outpatient setting.^13^ Nearly all cancer medicines used in Australia are PBS-listed, including cytotoxic chemotherapy, molecularly targeted therapies, and immune checkpoint inhibitors.

PBS dispensing claims are processed and maintained by Services Australia. These data are complete records of medicines dispensed in the community in Australia. We used the PBS 10% sample dataset—a standard dataset provided by Services Australia that includes all prescription medicine claims for a nationally-representative, random 10% sample of the PBS-eligible population (all Australian citizens and permanent residents). These de-identified, individual-level data include the name of the dispensed medicine, date of dispensing, a PBS-specific item code that denotes the intended treatment indication, and the recipient’s sex, year of birth, and year of death.^13^ We used dispensing records for all cancer medicines from March 2005, when the PBS 10% sample data series commenced, through December 2021 to determine the number of people with exposure to potentially cardiotoxic cancer medicines alive during each calendar year. Where a medicine was indicated to treat both malignancies and non-malignant conditions, we used PBS item codes to exclude dispensings intended for the latter.

### Cardiovascular toxicities and medicines of interest

The cardiovascular toxicities we included were: sudden cardiac death; left ventricular dysfunction and heart failure (symptomatic and asymptomatic); arrhythmias and QT prolongation; ischaemia and myocardial infarction; coronary vasospasm; takotsubo syndrome; myocarditis, pericarditis, pericardial effusion, and tamponade; hypertension and hypotension; hyperlipidaemia; hyperglycaemia; arterial thromboembolism; venous thromboembolism; pulmonary hypertension; and vasculitis. Our definition of left ventricular dysfunction includes decline in ejection fraction and cardiomyopathy. We did not apply a specific threshold for ejection fraction or decline in ejection fraction.

We reviewed the published academic and grey literature to identify a comprehensive list of cancer medicines (i.e., chemotherapies, endocrine therapies, targeted therapies, and immunotherapies) with evidence of association with these toxicities. The most current and comprehensive list of potentially cardiotoxic cancer medicines was the European Society of Medical Oncology’s (ESMO) consensus recommendations, published in 2020.^14^ We adapted the ESMO list for the Australian context by removing 12 medicines that were not subsidised through the PBS during the study period and adding 42 medicines following a review of Product Information (PI) documents for all cancer medicines listed on the PBS,^15^ as defined by using World Health Organisation Anatomical Therapeutic Chemical (ATC) codes (ATC codes beginning, “L01” and “L02”).^16^ PI documents list adverse effects from pre-clinical, clinical, and post-marketing reports and the grade and frequency of each toxicity. We used the CIOMS III convention^17^ for frequency of adverse effects reporting.

There were 28 commonly used, PBS-subsidised medicines that we considered *not* cardiotoxic: abemaciclib, afatinib, blinatumomab, brentuximab, carboplatin, chlorambucil, eribulin, fotemustine, fulvestrant, gefitinib, hydroxyurea, idelalisib, inotuzumab ozagamicin, irinotecan, mercaptupurine, methotrexate, olaparib, oxaliplatin, palbociclib, pemetrexed, pralatrexate, raltitrexed, sonidegib, temozolomide, tioguanine, topotecan, venetoclax, and vismodegib. The final list contained 99 potentially cardiotoxic cancer medicines (Supplementary Tables A and B). Non-cancer indications for these medicines were identified by PBS item code and excluded from the study.

### Study population and statistical analyses

We identified all people dispensed at least one potentially cardiotoxic cancer medicine during the study period. We calculated annual prevalence rates per 10,000 population as the number of people alive with exposure to a potentially cardiotoxic medicine during or prior to each year of the study period (numerator) over the Australian Bureau of Statistics (ABS) estimated resident population (divided by 10; denominator),^18^ and then used the ABS standard population for direct age-standardisation with 10-year age groups.^19^^,^^20^ If a person’s year of death was the same as their year of exposure, they were considered alive during that year and appear in the numerator for that year. We calculated prevalence rates overall; rates for exposure to medicines with expected cardiovascular toxicity frequency of >1%; by the number of years alive following exposure (five and 10 years following first exposure to a medicine of interest); and by the number of medicines interest a person was exposed to (1, 2, or 3 or more medicines over the study period, concurrently or otherwise). We further stratified each of these outcomes by sex. We also calculated age-specific overall prevalence rates. As a sensitivity analysis we performed all analyses excluding tamoxifen, anastrazole, letrozole and exemestane as the net effect of these agents is debateable. There is current evidence suggesting a neutral or cardioprotective effect of tamoxifen alongside its increased risk of vascular toxicity, and most of the evidence of cardiotoxicity of aromatase inhibitors (AIs) is derived from trials comparing to Tamoxifen.^21^^,^^22^

We performed all analyses in R version 4.3 and used ggplot2 to generate all figures.^23^^,^^24^

### Ethics and data access

The NSW Population & Health Services Research Ethics Committee granted ethics approval (2019ETH1176) and the Services Australia External Request Evaluation Committee approved data access (MI8157, RMS1941). Access to the data and analytical files used in our study by other individuals or authorities is not permitted without the express permission of the approving human research ethics committees and data custodians.

### Role of the funding source

The funders had no role in the research.

## Results

Of the 198,352 people dispensed any cancer medicine between 2005 and 2021, we observed 108,175 (55%) dispensed at least one potentially cardiotoxic cancer medicine. They were alive and previously exposed to potentially cardiotoxic medicines for a median of 4 years (mean = 5.8 years). Their median age at the time of first dispensing was 64 (IQR: 52–74); 57% were female; and 39% died during the study period.

**Fig. 1:**
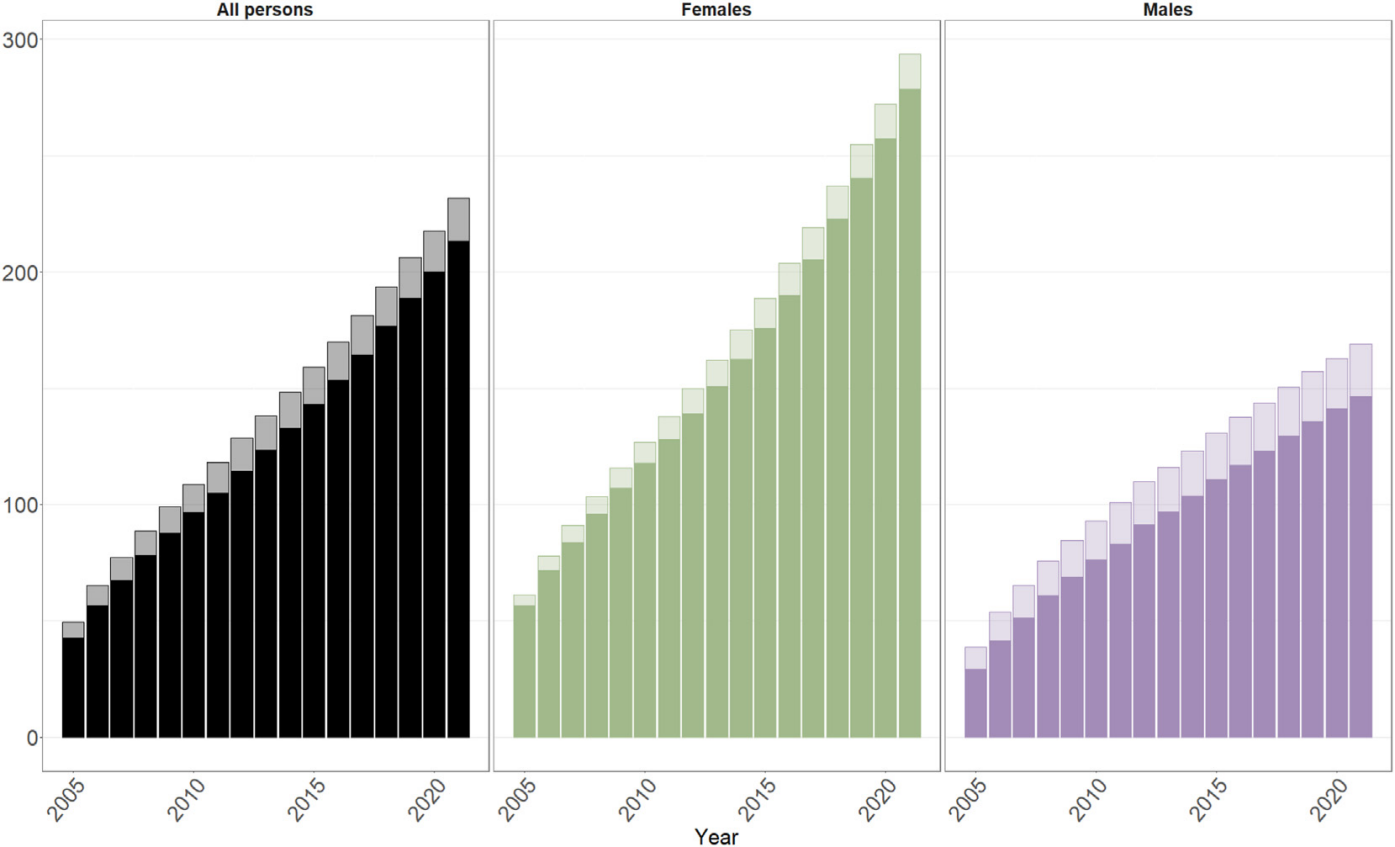
Age-standardised rates (per 10,000) of people with previous exposure to any potentially cardiotoxic pharmaceutical cancer medicine (lighter shading) and those medicines with more common frequency of cardiotoxicity (>1% prevalence; darker shading) and alive during each year, 2005–2021 (inclusive). All persons and stratified by patient sex.

The overall prevalence of exposure to potentially cardiotoxic medicines more than quadrupled from 49 (95%CI: 48.7–49.3)/10,000 in 2005 to 232 (95%CI: 231.4–232.6)/10,000 in 2021 for all people; 61 (95%CI: 60.5–61.5)/10,000 to 293 (95%CI: 292.1–293.9)/10,000 for females; and 39 (95%CI: 38.6–39.4)/10,000 to 169 (95%CI: 168.3–169.7)/10,000 for males; with no evidence of a plateau (Fig. 1 and Supplementary Table C). Overall prevalence of exposure to medicines with an expected cardiovascular toxicity frequency of >1% similarly increased from 43 (95%CI: 42.7–43.3)/10,000 in 2005 to 213 (95%CI: 212.5–213.5) for all people; 57 (95%CI: 56.6–57.4)/10,000 to 278 (95%CI: 277.1–278.9)/10,000 for females; and 29 (95%CI: 28.7–29.3)/10,000 to 146 (95%CI: 145.4–146.6)/10,000 for males.

**Fig. 2:**
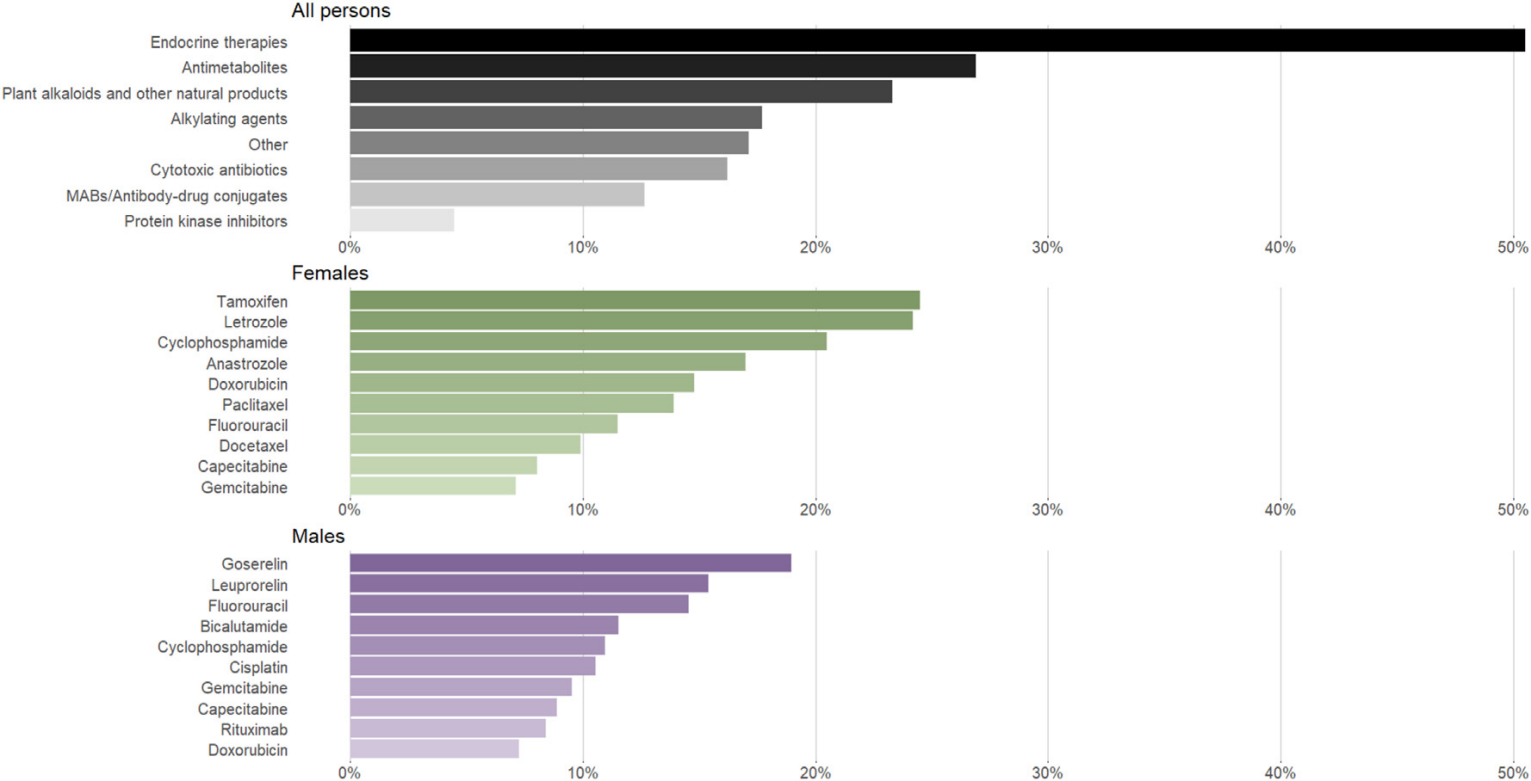
Proportion of the cohort dispensed at least one medicine from each ATC grouping (top panel); and top 10 potentially cardiotoxic cancer medicine dispensed to the largest proportions of females (middle panel) and males (bottom panel).

**Fig. 3:**
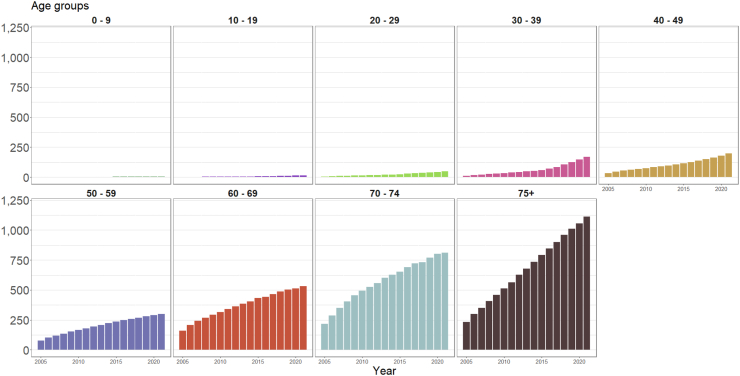
Age-specific rates (per 10,000) of the number of people with previous exposure to potentially cardiotoxic pharmaceutical cancer medicines and alive during each year, 2005–2021 (inclusive).

The largest proportions of the cohort were exposed to endocrine therapies (e.g., anastrozole, tamoxifen; dispensed to 51% of the cohort), antimetabolites (e.g., capecitabine, pemetrexed; 27%), and plant alkaloids and other natural products (e.g., taxanes, irinotecan; 23%; Fig. 2). The largest proportions of females were dispensed tamoxifen (25%), letrozole (24%), and cyclophosphamide (21%); and the largest proportions of males were dispensed goserelin (18%), leuprorelin (15%), and fluorouracil (15%; Fig. 2). Exposure rates were higher in older age groups than younger age groups (Fig. 3 and Supplementary Table D).

**Fig. 4:**
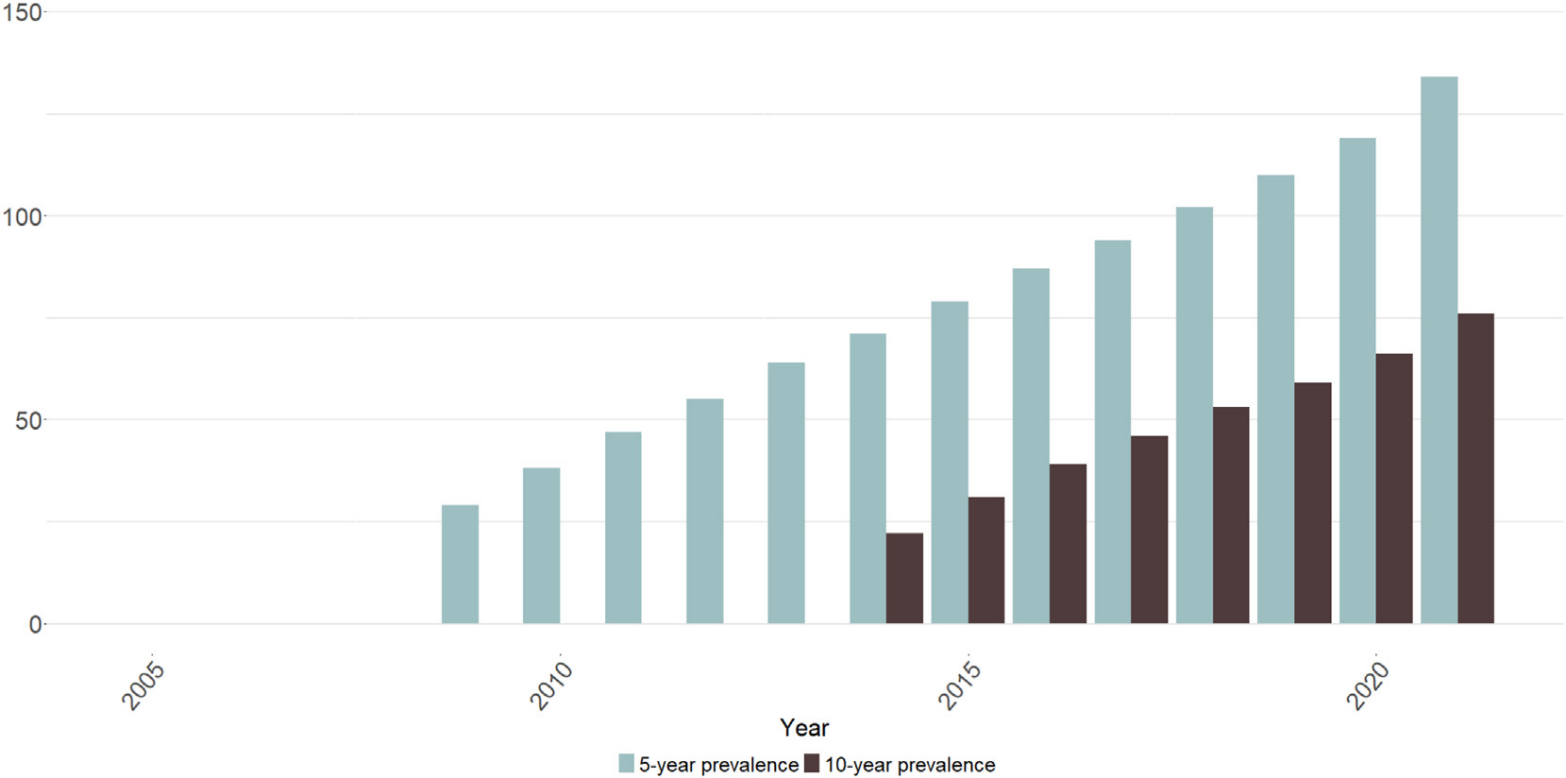
Age-standardised rates (per 10,000) of the number of people with previous exposure to potentially cardiotoxic pharmaceutical cancer medicines and alive during each year after N years (i.e., 1, 5, and 10 years), 2005–2021 (inclusive).

**Fig. 5:**
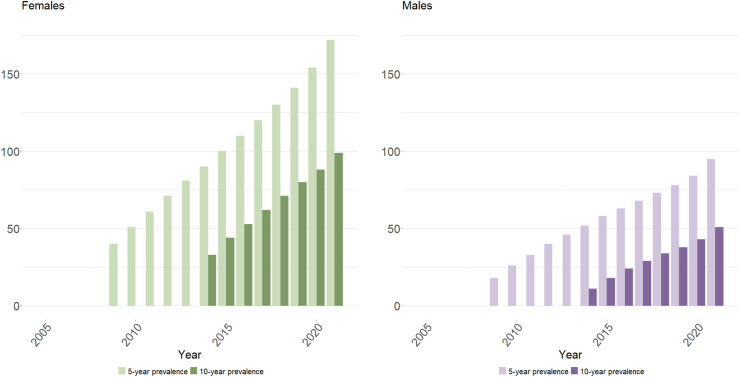
Age-standardised rates (per 10,000) of the number of people with previous exposure to potentially cardiotoxic pharmaceutical cancer medicines and alive during each year after 5 and 10 years; stratified by sex, 2005–2021 (inclusive).

Prevalence rates of people alive at least five years following first exposure to potentially cardiotoxic cancer medicines increased from 29 (95%CI: 28.8–29.2)/10,000 in 2009 to 134 (95%CI: 133.6–134.4)/10,000 in 2021 (Fig. 4 and Supplementary Table E). Rates for the number of people alive at least 10 years following first exposure increased from 22 (95%CI: 21.8–22.2)/10,000 in 2014 to 76 (95%CI: 75.7–76.3)/10,000 in 2021. Five- and 10-year rates stratified by sex followed a similar pattern, with rates for females increasing from 40 (95%CI: 39.6–40.4)/10,000 to 172 (95%CI: 171.3–172.7)/10,000 and 33 (95%CI: 32.7–33.3)/10,000 to 99 (95%CI: 98.5–99.5)/10,000, respectively. Five- and 10-year prevalence rates for males increased from 18 (95%CI: 17.7–18.3)/10,000 to 95 (95%CI: 94.5–95.5)/10,000 and 11 (95%CI: 10.8–11.2)/10,000 to 51 (95%CI: 50.6–51.4)/10,000, respectively (Fig. 5 and Supplementary Table E).

**Fig. 6:**
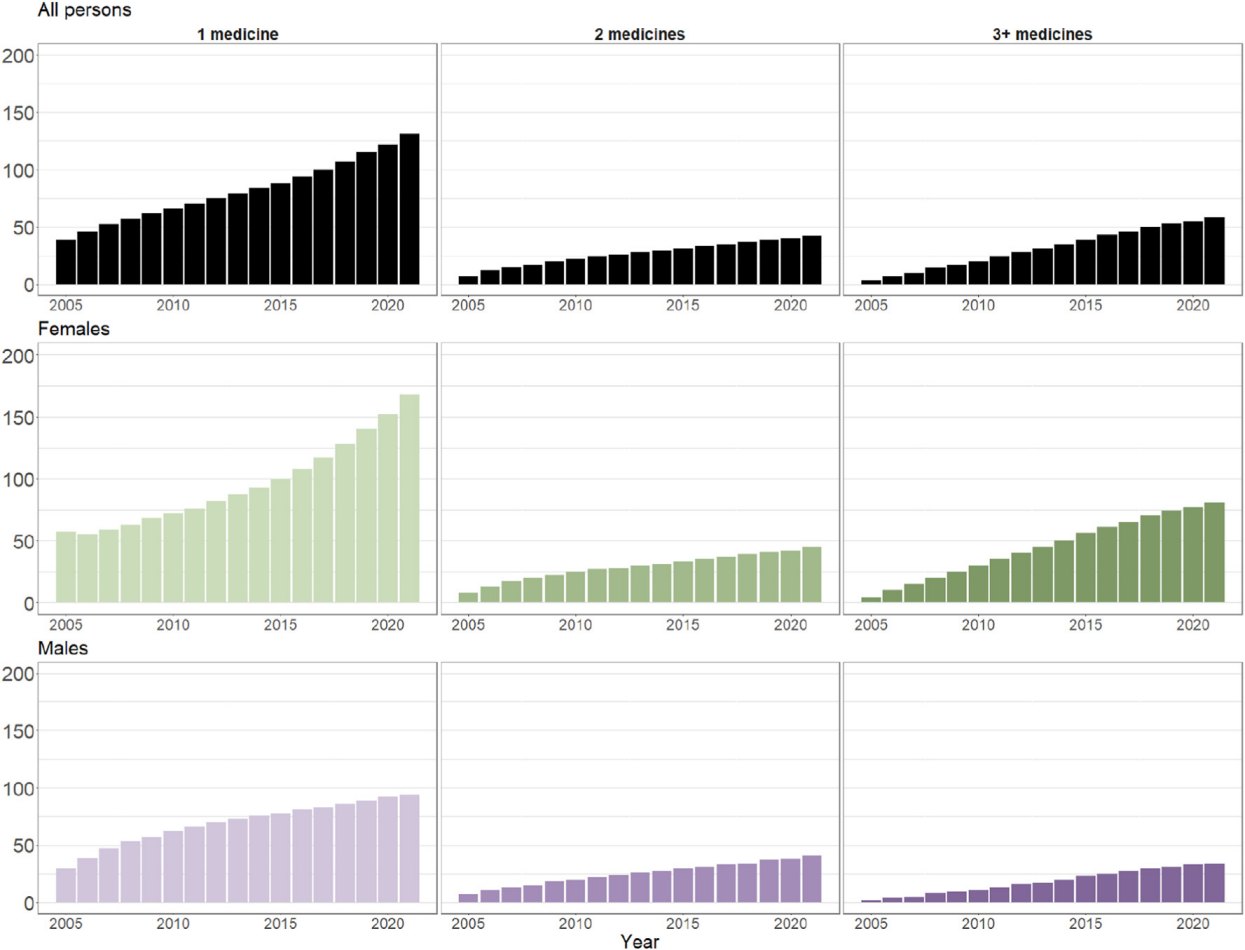
Age-standardised rates (per 10,000) of the number of people with previous exposure to potentially cardiotoxic pharmaceutical cancer medicines and alive during each year, 2005–2021 (inclusive). Stratified by the number of potentially cardiotoxic medicines a patient was exposed to by that year (i.e., a patient exposed to one medicine in 2005 would be counted as ‘1 medicine’ until they were exposed to another medicine, after which point they would be counted as ‘2 medicines’) and sex.

Most people were exposed to only one potentially cardiotoxic medicine, with rates increasing from 39 (95%CI: 38.7–39.3)/10,000 in 2005 to 131 (95%CI: 130.6–131.4)/10,000 in 2021 (Fig. 6 and Supplementary Table F). Prevalence rates increased from 7 (95%CI: 6.9–7.1)/10,000 to 42 (95%CI: 41.8–42.2)/10,000 for people exposed to two different potentially cardiotoxic medicines and 3 (95%CI: 2.9–3.1)/10,000 to 58 (95%CI: 57.7–58.3)/10,000 for those exposed to three or more potentially cardiotoxic medicines.

Females experienced higher rates of potentially cardiotoxic medicine exposure than their male counterparts, driven by dispensings of common breast cancer therapies, e.g., endocrine therapies, anthracyclines, and taxanes. Females with exposure to three or more medicines were more prevalent than females with exposure to two medicines; while males with exposure to two medicines were slightly more prevalent than those with exposure to three or more medicines (Fig. 6 and Supplementary Table F).

The prevalence of people alive at least five and 10 years following first exposure to one, two, or three or more potentially cardiotoxic cancer medicines followed similar increasing patterns to those from each outcome individually (Supplementary Fig. A and Table G). Most people have exposure to just one medicine, with five- and 10-year prevalence rates peaking at 71 (95%CI: 70.6–71.4) and 40 (95%CI: 39.6–40.4)/10,000, respectively, in 2021. Five- and 10-year prevalence rates of people exposed to three or more medicines were slightly higher than those of people exposed to two medicines. Prevalence rates were higher for females in all stratifications (Supplementary Fig. B and Table G).

Prevalence rates from the sensitivity analyses were roughly 25% lower than in the main analyses. The rates for females decreased more than those for males, and between 2005 and 2016, rates of exposure to potentially cardiotoxic cancer medicines were higher in men. Prevalence rates of those exposed to potentially cardiotoxic cancer medicines and alive five and 10 years from first exposure were roughly 35% lower than in the main analyses and remained higher in women. Prevalence rates stratified by the number medicines a person was exposed to were roughly 35% lower than in the main analyses; females and males had similar rates of exposure to one and two potentially cardiotoxic cancer medicines, while rates for three or more medicines were higher in women (Supplementary Tables H through L).

## Discussion

Our study demonstrates that the overall burden of exposure to all potentially cardiotoxic cancer medicines, and those with an expected frequency of >1%, is substantial and growing, with no evidence of stabilising over the past two decades. While over half of the people in our study were exposed to potentially cardiotoxic medicines, females were disproportionately affected, driven largely by exposure to pharmacological treatments for breast cancer. Rates of survivorship also increased during the study period,^25^ meaning that higher rates of people exposed to potentially cardiotoxic cancer medicines are living beyond the time of initial exposure.

To our knowledge, ours is the first study to attempt to quantify the extent of exposure to potentially cardiotoxic medicines in the Australian population, and our findings can assist in guiding future health services planning as well as future research exploring the safety of cancer medicines. Extrapolating our findings from a 10% sample to the wider Australian population suggests that between 2005 and 2021 over one million Australians were exposed to potentially cardiotoxic cancer medicines. Cancer is a disease of advanced age and, unsurprisingly, we found rates of exposure to potentially cardiotoxic cancer medicines highest in older age groups. As a large proportion of the Australian population has or will soon enter the age groups at highest risk of a cancer diagnosis, rates of exposure to potentially cardiotoxic cancer medicines are likely to rise further in the coming decades. The future integration of multiple population-based datasets will allow estimation of incident cardiovascular events and their determinants.

When tamoxifen and AIs were excluded from the analyses, the exposure rates in women were closer to those in men. These potentially cardiotoxic cancer medicines were amongst the most frequently dispensed to females in our study, and they are used predominantly in women. The extent of cardiovascular toxicity from tamoxifen and AIs is not clear cut. The weight of evidence suggests that tamoxifen is cardioprotective or neutral, but may increase risks of thromboembolism 2^21^^,^^22^; while AIs are associated with hyperlipidaemia and increased risks of cardiovascular disease.^26^ However, the evidence supporting these increased risks associated with AIs has been largely derived from studies comparing aromatase inhibitors to tamoxifen—meaning that differences in observed cardiovascular outcomes are potentially driven by cardioprotective effects of tamoxifen.^21^ Longer durations of AI treatment (e.g., extended adjuvant therapy) are associated with increased risks of cardiovascular events compared to placebo.^27^ Cardiovascular risk is an important consideration for breast cancer survivors, who may be at higher risk of events due to previous treatment (e.g., radiotherapy, prior anthracycline and/or trastuzumab treatment) and close monitoring is recommended during endocrine therapy.^12^^,^^28^

Survival outcomes improved and new medicines—many of which are potentially cardiotoxic—were continually introduced to the market during the study period. Our finding that prevalence rates for patients exposed to three or more medicines were higher than those for patients exposed to two potentially cardiotoxic cancer medicines does not imply a causal relationship and one should not be inferred. There are numerous unmeasured factors behind these results, including that patients with longer survival have more opportunity to become exposed to more potentially cardiotoxic medicines. That, generally, the prevalence of exposure to potentially cardiotoxic medicines increased each year of the study period likely reflects both improved survival and more treatment options and underscores the need for more research into the real-world safety outcomes as more people are likely to be treated with a more diverse range of medicines in the decade ahead.

The occurrence of cardiac disease in people treated pharmacologically for cancer is multifactorial and, typically, not solely dependent on the medicine itself. Cardiotoxicity can also occur as a result of radiotherapy exposure.^29^ Preventive health care, early detection, risk stratification prior to cancer treatment, and proactive management of cardiovascular insults are vital. Cardio-oncology is a relatively new and evolving medical specialty, and there has been significant variation in the terminology and definitions used in this area to date. A new set of guidelines and quality indicators published in 2022 comprise a significant step towards helping institutions implement recommended care, reduce variation in that care, and quantify the quality of cancer treatment-related cardiac care.^12^^,^^30^ The guidelines note that clearly specified roles for different health professionals (e.g., nurses, specialists) are still needed, and we agree with previous calls for greater integration of primary care practitioners in cardio-oncology care.^31^

The complexity of complications from cardiovascular toxicities can be devastating for patients. Cardiotoxicity may require cessation of treatment and/or the limiting of potential therapies, and patients may survive the cancer only to suffer ongoing health issues from the treatment. These patients may also face long-term health care costs, adding to their emotional and economic burden. Longer-term impacts are significant, as our findings suggest a substantial proportion of patients exposed to these medicines are surviving at least five years from the time of first exposure. Patients need clear communication detailing how pharmaceutical cancer treatments may harm the cardiovascular system, particularly when undergoing multiple therapies.

### Strengths and limitations

Our study used large, nationally-representative data comprising dispensing records for 10% of the PBS-eligible Australian population observed over nearly two decades. These data do not contain clinical information, nor do we have information about the development of cardiovascular disease in our cohort. Our study is, therefore, descriptive and an examination of the factors associated with exposure to potentially cardiotoxic cancer medicines is beyond the scope of the current work. Most pharmacological cancer treatments are subsidised by the PBS and, therefore, appear in our data. A small number of treatments may have been dispensed to public hospital inpatients, patients paying for medicines out of pocket, and those accessing medicines through compassionate access schemes or clinical trials. These treatments are not captured in our data and our results likely under-estimate the true rates of exposure to potentially cardiotoxic cancer medicines. Similarly, prior to 2012 PBS dispensing records did not capture medicines costing less than the PBS-copayment threshold.^13^ Most cancer medicines cost well above PBS co-payment thresholds in each year of our study, however, the cost of some formulations of tamoxifen and cyclophosphamide were below co-payment thresholds between 2005 and 2012 and our results may under-estimate the true rates of exposure to these medicines during this period.

Over the past two decades, the definition of treatment-induced cardiac toxicity has been a narrow one, based largely around myocardial dysfunction associated with anthracyclines and HER2-targeted therapies. In recent years, definitions have expanded to include symptomatic and asymptomatic cardiac dysfunction, myocarditis, arterial hypertension and other vascular effects, and conduction system abnormalities.^5^^,^^7^^,^^14^^,^^32^ Some medicines may be more or less cardiotoxic than they appear in current evidence and clinical trials may underestimate the real-world occurrence of cardiac disease, as people at greatest risk of cardiac disease are excluded or the trial population is not followed up for the duration required to observe the event.

In our study, medicines with evidence of cardiovascular toxicity were included regardless of frequency, duration or severity of these adverse effects, and the quality of evidence. Frequency does not equate to clinical significance and the frequency of adverse effects of many medicines varies across published studies, treatment settings, and published criteria used to define cardiac adverse events. Rare events may be critically significant, such as fulminant myocarditis associated with immune checkpoint inhibitor use; while minor arrhythmias, such as sinus bradycardia or tachycardia, occur frequently but have minimal long-term consequences or need for intervention. Conversely, toxicities such as moderate hypertension have little consequence in the short term but are important risk factors for development of cardiovascular disease over the course of persons life. There is subjective judgement around the classification of the cardiotoxicity of specific cancer medicines, with varying levels of evidence to support such classifications. Our list of potentially cardiotoxic cancer medicines is broad but amongst the most comprehensive in the published literature, and our results should be interpreted with this in mind.

The number of people exposed to potentially cardiotoxic cancer medicines in Australia is substantial and growing. Our findings can be used to develop service planning, guide future research, and create awareness of the magnitude of the issue of cancer treatment-related cardiotoxicities. The complexities of cardiovascular toxicities and increasing role of cardio-oncology necessitate co-operation between health care professionals to ensure delivery of care that identifies and mitigates the risk of complications. Ongoing monitoring and surveillance of both exposures and outcomes are necessary so that resources can be directed in a manner that is appropriate to the needs of the population.

## Contributors

Conceptualisation: All authors.

Data curation: BD.

Formal analysis: BD.

Funding acquisition: CV, SAP, BD.

Investigation: All authors.

Methodology: BD, MA, CV, SAP, MVL.

Project administration: BD, MA, CV, MVL.

Writing – original draft: BD, MA.

Writing – review & editing: All authors.

## Data sharing statement

Access to the data and analytical files used in our study by other individuals or authorities is not permitted without the express permission of the approving human research ethics committees and data custodians.

## Declaration of interests

BD: Support for the present manuscript (Cancer Institute NSW Early Career Fellowship; NHMRC Medicines Intelligence Centre of Research Excellence).

MA: no interests to declare.

MVL: Grants or contracts from any entity (National Health and Medical Research Council (NHMRC) Postdoctoral Fellowship 1012141).

MB: no interests to declare.

LH: no interests to declare.

HG: Participation on a Data Safety Monitoring Board or Advisory Board (BMS, Pfizer, MSD, Merck Serono, Ipsen, Roche, Astellas).

MT: no interests to declare.

SAP: Support for the present manuscript (NHMRC Medicines Intelligence Centre of Research Excellence).

CMV: Support for the present manuscript (NHMRC Medicines Intelligence Centre of Research Excellence).
