# Supplementary material for: Prevalence of Australians exposed to potentially cardiotoxic cancer medicines: a population-based cohort study
Source: Lancet Reg Health West Pac. 2023 Aug 2;39:100872. doi: 10.1016/j.lanwpc.2023.100872 (PMC10410507; doi:10.1016/j.lanwpc.2023.100872)
Supplement: Supplementary Tables — A–L [file mmc1.docx]

Supplementary Table A. Potentially cardiotoxic medicines of interest per ESMO publication

This list has been adapted with permission from:

Curigliano G, Lenihan D, Fradley M, Ganatra S, Barac A, Blaes A, et al. Management of cardiac disease in cancer patients throughout oncological treatment: ESMO consensus recommendations. Ann Oncol. 2020;31(2):171-90. Supplementary Table S1, Anticancer therapies associated with CV complications or toxicities.

**Medicines from this list that were not subsidised through the PBS during the study period are in grey font. We did not include these medicines in our analysis as we cannot ascertain their use in Australia.**

| **Anticancer agents** | **Cancer use** | **Type of cardiotoxicity** | **Frequency** |
| --- | --- | --- | --- |
| **Anthracyclines** | | | |
| Doxorubicin [1, 2] | Breast, sarcoma,  lung, bladder,  gastric,  prostate,  leukaemia,  lymphoma,  others | HF  LVD  Arrhythmia | Common |
| Epirubicin [3] | Breast,  oesophageal,  gastric | HF  LVD  Arrhythmia | Common |
| **Alkylating agents** | | | |
| Cyclophosphamide [4] | Breast,  lymphoma,  myeloma,  sarcoma, SCT | HF  LVD  Myopericarditis  Arrhythmia | Uncommon |
| Ifosfamide [5, 6] | Testicular,  sarcoma,  lymphoma | HF  LVD  Myopericarditis  Arrhythmia | Common |
| Cisplatin [7, 8] | Lung, bladder,  testicular,  breast,  oesophageal,  head and  neck | Arrhythmia  Ischaemia  VTE  HTN | Uncommon  Common |
| Melphalan [9, 10] | MM, ovarian,  neuroblastoma,  SCT | Arrhythmia  LVD | Common  Rare |
| **Antimetabolites** | | | |
| Fluorouracil [11-15] | Colon, pancreatic,  breast, head and  neck | Coronary  vasospasm  Ischaemia  Arrhythmia  LVD  Myocarditis | Common  Uncommon  Rare |
| Capecitabine [16, 17] | Breast, colon,  gastric,  pancreatic | Coronary  vasospasm  Ischaemia  Arrhythmia  LVD | Common  Uncommon |
| Fludarabine [18, 19] | Lymphoma,  leukaemia, SCT | Angina  Ischaemia  Arrhythmia  LVD | Uncommon |
| Decitabine [20] | MDS | HF  LVD | Uncommon |
| **Antimicrotubule agents** | | | |
| Docetaxel [21, 22] | Breast, lung, prostate,  gastric, head and  neck | HF  LVD  Arrhythmia | Uncommon |
| Paclitaxel [23-25] | Breast, ovarian, lung,  sarcoma, bladder,  cervical, gastric,  oesophageal,  head and neck | Ischaemia  Bradyarrhythmia | Rare |
| Vinblastine [26-28] | Lymphoma, testicular,  lung, melanoma | Ischaemia  Arrhythmia | Rare |
| **Monoclonal antibodies** | | | |
| Rituximab [29]  Ofatumumab [30]  Alemtuzumab [31] | Lymphoma,  leukaemia | Hypotension  (infusion  reaction)  HTN  LVD  HF | Common  Uncommon |
| **Monoclonal antibodies (HER2)** | | | |
| Bevacizumab [32-34] | Colorectal, cervical,  glioblastoma,  ovarian, renal,  endometrial,  sarcoma, breast | HTN  VTE  ATE  Myocardial  ischaemia  LVD | Common  Uncommon |
| Pertuzumab [35] | Breast | HF  LVD | Uncommon |
| Trastuzumab [36-38] | Breast, gastric,  gastroesophageal | HF  LVD | Common |
| **Small-molecule TKIs** | | | |
| Dabrafenib [39-41] | Melanoma | QT prolongation  HF  LVD  VTE | Common  Rare^a^ |
| Dasatinib [42, 43] | Leukaemia, GIST | HF  LVD  QT prolongation  Pulmonary HTN | Uncommon  Rare |
| Lapatinib [44-46] | Breast | HF  LVD | Uncommon |
| Pazopanib [47, 48] | Renal, sarcoma,  thyroid | HTN  Bradyarrhythmia  HF  LVD  VTE  QT prolongation  ischaemia | Common  Uncommon |
| Ponatinib [42, 49, 50] | Leukaemia | HF  LVD  HTN  Ischaemia  ATE  VTE | Common |
| Sorafenib [51-54] | Hepatocellular, renal,  thyroid | HTN  HF  LVD  Ischaemia | Common |
| Trametinib [40, 55, 56] | Melanoma | HF  LVD  Bradyarrhythmia  QT prolongation  VTE  HTN | Common |
| Sunitinib [34, 48, 57] | Renal, thyroid,  sarcoma, GIST,  PNET | HTN  HF  LVD  VTE  ATE | Common  Uncommon |
| Axitinib [58] | Renal | HTN  VTE | Common  Uncommon |
| Nilotinib [42, 59] | Leukaemia | Ischaemia  VTE  ATE  QT prolongation  Atherosclerosis  Hyperglycaemia/  diabetes | Uncommon |
| Ibrutinib [60, 61] | Lymphoma | Atrial fibrillation  HTN  Bleeding  Ventricular  arrhythmia | Common  Rare |
| Ramucirumab [62, 63] | Colorectal, gastric,  lung | HTN  ATE  VTE | Common  Uncommon |
| Regorafenib [63, 64] | Colorectal, GIST | HTN  Ischaemia | Common  Rare |
| Imatinib [42, 65] | Leukaemia, GIST,  MDS, melanoma,  mastocytosis,  sarcoma | LVD  HF  Oedema | Rare  Common |
| Vandetanib [63, 66] | Thyroid | HTN  QT prolongation  HF  LVD | Common  Rare |
| Ziv-aflibercept [63, 67] | Colorectal cancer | HTN  QT prolongation  VTE  ATE | Common  Uncommon |
| Cabozantinib [63, 68] | Thyroid, renal | HTN  VTE | Common |
| Erlotinib [69-71]  Cetuximab [72, 73] | Lung, pancreatic,  colorectal | VTE  Ischaemia  SCD | Common  Rare |
| Ceritinib [74] | Lung | QT prolongation  Bradyarrhythmia | Uncommon |
| Crizotinib [75] | Lung | Bradyarrhythmia^b^  QT prolongation | Uncommon |
| Vemurafenib [41, 76] | Melanoma | HTN  QT prolongation  Arrhythmia  VTE | Uncommon  Rare |
| **Immune checkpoint inhibitors** | | | |
| Nivolumab  Ipilimumab  Pembrolizumab  [77-79] | Melanoma, lung,  kidney, bladder,  head and neck,  lymphoma | Myocarditis  Arrhythmia  LVD  SCD  Vasculitis  Pericarditis | Uncommon |
| **Protease inhibitors** | | | |
| Bortezomib [80, 81] | MM, MCL | HF  LVD  VTE^c^  HTN | Uncommon  Common |
| Carfilzomib [82-84] | MM | HF  LVD  VTE  HTN  ACS  Pulmonary HTN | Common  Uncommon |
| **mTOR inhibitors** | | | |
| Everolimus [85] | Breast, pancreas | HTN  VTE | Common  Uncommon |
| Temsirolimus [86] | Renal | HTN | Common |
| **IMiDs** | | | |
| Lenalidomide [87]  Thalidomide [88]  Pomalidomide [89] | MM | VTE  Bradycardia | Common  Uncommon |
| **Histone deacetylase inhibitors** | | | |
| Vorinostat [90, 91]  Belinostat [92] | Lymphoma | QT prolongation  VTE | Common  Uncommon |
| **Endocrine therapy** | | | |
| Selective ER  modulators   - Tamoxifen [93] - Toremifene [94] | Breast cancer | VTE  QT prolongation | Common |
| AIs   - Anastrozole [95] - Letrozole [96] - Exemestane [97] | Breast cancer | VTE  HTN  Hyperlipidaemia | Common |
| LHRH agonists   - Goserelin [98] - Leuprolide [99] | Breast, endometrial, prostate | Ischaemia  VTE  CVA  HF  LVD  QT prolongation | Uncommon |
| Antiandrogens   - Flutamide [100] - Bicalutamide [101] - Nilutamide [102] | Prostate cancer | Arrhythmia  ATE  VTE  HF  LVD  QT prolongation  HTN | Uncommon  Common |
| **Chimeric antigen receptor (CAR) T Cell Therapy** | | | |
| Tisagenlecleucel [103]  Axicabtagene ciloleucel [104] | B cell acute lymphoblastic leukaemia (refractory or relapse)  Large B cell lymphoma (refractory or relapse) | Tachycardia  Arrhythmia  Hypotension  HTN  HF  Capillary leak syndrome  MI (unrelated to CAD, likely due to antigen mimicry)  Cardiac arrest | Common  Uncommon |
| **Miscellaneous** | | | |
| Ribociclib [105] | Breast cancer | QT prolongation | Uncommon^d^ |
| Bleomycin [106-108] | SCC, melanoma, sarcoma, testicular, lymphoma | Ischaemia  Pericarditis  CVA | Rare |
| Tretinoin [109, 110] | Leukaemia | HF  LVD | Common |
| Arsenic trioxide [111, 112] | Leukaemia | QT prolongation  Heart block | Common  Rare |

^a^These side effects are common when used in combination with trametinib.

^b^Per package insert, it was reported in 5 % of cases but was of low grade.

^c^VTE is common with bortezomib if used in combination with IMiDs.

^d^Clinical trials excluded patients on other QT prolonging medications. In the clinical setting, this might be more common due to concomitant use of other QT-prolonging medications.

**The frequency of toxicity was graded as**: common ≥5% incidence, uncommon 1%–5% incidence or rare <1% incidence in clinical trials or observational studies.

ACS, acute coronary syndrome; AI, aromatase inhibitor; ATE, arterial thromboembolism; CAD, coronary artery disease; CV, cardiovascular; CVA, cerebrovascular accident; ER, oestrogen receptor; GIST, gastrointestinal stromal tumour; HER2, human epidermal growth factor receptor 2; HF, heart failure; HTN, hypertension; IMiD, immunomodulatory drug; LHRH, luteinizing hormone-releasing hormone; LVD, left ventricular dysfunction; MCL, mantle cell lymphoma; MDS, myelodysplastic syndrome; MI, myocardial infarction; MM, multiple myeloma; mTOR, mammalian target of rapamycin; PNET, pancreatic neuroendocrine tumour; QT, corrected QT interval (preferably by Fridericia’s formula); SCC, squamous cell cancer; SCD, sudden cardiac death; SCT, stem cell transplant; TKI, tyrosine kinase inhibitor; VTE, venous thromboembolism.

**References for Suppplementary Table A**

1. Doxorubicin Hydrochloride for Injection, USP [package insert]. Phramacia & Upjohn Company, Division of Pfizer Inc, NY, NY 10017. 2010.

2. Murbraech K, Wethal T, Smeland KB et al. Valvular Dysfunction in Lymphoma Survivors Treated With Autologous Stem Cell Transplantation: A National Cross-Sectional Study. JACC Cardiovasc Imaging 2016; 9: 230-239.

3. Epirubicin Hydrochloride for Injection [package insert]. Mayne Pharma Limited Mulgrave, VIC 3170, Australia. 2006.

4. Cyclophosphamide [package insert]. Baxter Healthcare Corporation Deerfield, IL 60015 USA. 2013.

5. Quezado ZM, Wilson WH, Cunnion RE et al. High-dose ifosfamide is associated with severe, reversible cardiac dysfunction. Ann Intern Med 1993; 118: 31-36.

6. Pai VB, Nahata MC. Cardiotoxicity of chemotherapeutic agents: incidence, treatment and prevention. Drug Saf 2000; 22: 263-302.

7. Cisplatin (Platinol) [package insert]. Bristol-Myers Squibb Company Princeton, New Jersey 08543 USA. 2010.

8. Czaykowski PM, Moore MJ, Tannock IF. High risk of vascular events in patients with urothelial transitional cell carcinoma treated with cisplatin based chemotherapy. J Urol 1998; 160: 2021-2024.

9. Yanamandra U, Gupta S, Khadwal A, Malhotra P. Melphalan-induced cardiotoxicity: ventricular arrhythmias. BMJ Case Rep 2016; 2016.

10. Melphalan (Alkeran) [package insert]. GlaxoSmithKline, Research Triangle Park, NC 27709.

11. Fluorouracil [package insert]. Spectrum Pharmaceuticals, Inc. Irvine, CA 92618. 2016.

12. Meyer CC, Calis KA, Burke LB et al. Symptomatic cardiotoxicity associated with 5-fluorouracil. Pharmacotherapy 1997; 17: 729-736.

13. Yeh ET, Bickford CL. Cardiovascular complications of cancer therapy: incidence, pathogenesis, diagnosis, and management. J Am Coll Cardiol 2009; 53: 2231-2247.

14. Sasson Z, Morgan CD, Wang B et al. 5-Fluorouracil related toxic myocarditis: case reports and pathological confirmation. Can J Cardiol 1994; 10: 861-864.

15. Killu A, Madhavan M, Prasad K, Prasad A. 5-fluorouracil induced pericarditis. BMJ Case Rep 2011; 2011.

16. Van Cutsem E, Hoff PM, Blum JL et al. Incidence of cardiotoxicity with the oral fluoropyrimidine capecitabine is typical of that reported with 5-fluorouracil. Ann Oncol 2002; 13: 484-485.

17. Capecitabine (Xeloda) [package insert]. Genentech USA, Inc. A Member of the Roche Group. 1 DNA Way, South San Francisco CA. 2015.

18. Fludarabine (Fludara) [package insert]. Berlex, Montville, NJ 07045. 2003.

19. Hussein MA, Gundacker H, Head DR et al. Cyclophosphamide followed by fludarabine for untreated chronic lymphocytic leukemia: a phase II SWOG TRIAL 9706. Leukemia 2005; 19: 1880-1886.

20. Decitabine (Dacogen) [package insert]. MGI PHARMA, INC., Bloomington, MN 55437. 2006.

21. Martin M, Pienkowski T, Mackey J et al. Adjuvant docetaxel for node-positive breast cancer. N Engl J Med 2005; 352: 2302-2313.

22. Docetaxel [package insert]. Sandoz, Princeton NJ 08540. 2012.

23. Paclitaxel (Taxol) [package insert]. Bristol-Myers Squibb Company, Princeton, NJ 08543 USA 2011.

24. Rowinsky EK, McGuire WP, Guarnieri T et al. Cardiac disturbances during the administration of taxol. J Clin Oncol 1991; 9: 1704-1712.

25. Arbuck SG, Strauss H, Rowinsky E et al. A reassessment of cardiac toxicity associated with Taxol. J Natl Cancer Inst Monogr 1993; 117-130.

26. Subar M, Muggia FM. Apparent myocardial ischemia associated with vinblastine administration. Cancer Treat Rep 1986; 70: 690-691.

27. Samuels BL, Vogelzang NJ, Kennedy BJ. Severe vascular toxicity associated with vinblastine, bleomycin, and cisplatin chemotherapy. Cancer Chemother Pharmacol 1987; 19: 253-256.

28. Vinblastine [package insert]. Bedford Laboratories, Bedford, Ohio 44146. 2012.

29. Rituximab [package insert]. Genentech, Inc. A Member of the Roche Group,1 DNA Way, South San Francisco, CA. 2010.

30. Ofatumumab (Arzerra) [package insert]. GlaxoSmithKline Research Triangle Park, NC 27709. 2009.

31. Alemtuzumab (Campath) [package insert]. Millennium and ILEX Partners, LP Cambridge, MA 02142. 2001.

32. Bevacizumab (Avastin) [package insert]. Genentech, Inc. 1 DNA Way, South San Francisco, CA. 2009.

33. Chen MH, Kerkela R, Force T. Mechanisms of cardiac dysfunction associated with tyrosine kinase inhibitor cancer therapeutics. Circulation 2008; 118: 84-95.

34. Li W, Croce K, Steensma DP et al. Vascular and Metabolic Implications of Novel Targeted Cancer Therapies: Focus on Kinase Inhibitors. J Am Coll Cardiol 2015; 66: 1160-1178.

35. Pertuzumab (Perjeta) [package insert]. Genentech, Inc. A Member of the Roche Group, 1 DNA Way, South San Francisco, CA. 2012.

36. Trastuzuman (Herceptin) [package insert]. Genentech, Inc. 1 DNA Way, South San Francisco, CA. 2010.

37. Slamon DJ, Leyland-Jones B, Shak S et al. Use of chemotherapy plus a monoclonal antibody against HER2 for metastatic breast cancer that overexpresses HER2. N Engl J Med 2001; 344: 783-792.

38. Suter TM, Procter M, van Veldhuisen DJ et al. Trastuzumab-associated cardiac adverse effects in the herceptin adjuvant trial. J Clin Oncol 2007; 25: 3859-3865.

39. Dabrafenib (Tafinlar) [package insert]. GlaxoSmithKline Research Triangle Park, NC 27709. 2014.

40. Flaherty KT, Infante JR, Daud A et al. Combined BRAF and MEK inhibition in melanoma with BRAF V600 mutations. N Engl J Med 2012; 367: 1694-1703.

41. Bronte E, Bronte G, Novo G et al. What links BRAF to the heart function? New insights from the cardiotoxicity of BRAF inhibitors in cancer treatment. Oncotarget 2015; 6: 35589-35601.

42. Moslehi JJ, Deininger M. Tyrosine Kinase Inhibitor-Associated Cardiovascular Toxicity in Chronic Myeloid Leukemia. J Clin Oncol 2015; 33: 4210-4218.

43. Dasatinib (Sprycel) [package insert]. Bristol-Myers Squibb Company, Princeton, NJ 08543 USA. 2010.

44. Perez EA, Koehler M, Byrne J et al. Cardiac safety of lapatinib: pooled analysis of 3689 patients enrolled in clinical trials. Mayo Clin Proc 2008; 83: 679-686.

45. Battisti NML, Tong D, Ring A, Smith I. Long-term outcome with targeted therapy in advanced/metastatic HER2-positive breast cancer: The Royal Marsden experience. Breast Cancer Res Treat 2019.

46. Lapatinib (Tykerb) [package insert]. GlaxoSmithKline, Research Triangle Park, NC 27709.

47. Pazopanib (Votrient) [package insert]. GlaxoSmithKline, Research Triangle Park, NC 27709. 2009.

48. Motzer RJ, Hutson TE, Cella D et al. Pazopanib versus sunitinib in metastatic renal-cell carcinoma. N Engl J Med 2013; 369: 722-731.

49. Ponatinib (Iclusig) [package insert]. ARIAD Pharmaceuticals, Inc. 26 Landsdowne Street, Cambridge, MA, USA. 2012.

50. Cortes JE, Kim DW, Pinilla-Ibarz J et al. A phase 2 trial of ponatinib in Philadelphia chromosome-positive leukemias. N Engl J Med 2013; 369: 1783-1796.

51. Sudasena D, Balanescu DV, Donisan T et al. Fulminant Vascular and Cardiac Toxicity Associated with Tyrosine Kinase Inhibitor Sorafenib. Cardiovasc Toxicol 2019; 19: 382-387.

52. Abdel-Rahman O, Fouad M. Risk of cardiovascular toxicities in patients with solid tumors treated with sorafenib: an updated systematic review and meta-analysis. Future Oncol 2014; 10: 1981-1992.

53. Pantaleo MA, Mandrioli A, Saponara M et al. Development of coronary artery stenosis in a patient with metastatic renal cell carcinoma treated with sorafenib. BMC Cancer 2012; 12: 231.

54. Sorafenib (Nexavar) [package insert]. Bayer HealthCare Pharmaceuticals Inc., Wayne, NJ 07470, USA. . 2010.

55. Trametinib (Mekinist) [package insert]. GlaxoSmithKline, Research Triangle Park, NC 27709, USA. 2014.

56. Flaherty KT, Robert C, Hersey P et al. Improved survival with MEK inhibition in BRAF-mutated melanoma. N Engl J Med 2012; 367: 107-114.

57. Sunitinib (Sutent) [package insert]. Pfizer Labs, Division of Pfizer Inc, New York, NY 10017, USA. 2011.

58. Axitinib (Inlyta) [package insert]. Pfizer Labs, Division of Pfizer Inc, New York, NY 10017, USA. 2012.

59. Nilotinib (Tasigna) [package insert]. Novartis Pharmaceuticals Corporation East Hanover, New Jersey 07936, USA. 2010.

60. Ibrutinib (Ibruvica) [package insert]. Pharmacyclics, Inc. Sunnyvale, CA USA 94085

2015.

61. Ganatra S, Majithia A, Shah S. Challenges in ibrutinib associated atrial fibrillation. J Am Coll Cardiol 2017; 11: 2308.

62. Ramucirumab (Cyramza) [package insert]. Eli Lilly and Company, Indianapolis, IN 46285, USA. 2014.

63. Santoni M, Guerra F, Conti A et al. Incidence and risk of cardiotoxicity in cancer patients treated with targeted therapies. Cancer Treat Rev 2017; 59: 123-131.

64. Regorafenib (Stivarga) [package insert]. Bayer HealthCare Pharmaceuticals Inc., Wayne, NJ 07470, USA. 2012.

65. Imatinib [package insert]. Novartis Pharmaceuticals Corporation, East Hanover, New Jersey 07936, USA. 2001.

66. Vandetanib (Caprelsa) [package insert]. AstraZeneca Pharmaceuticals LP, Wilmington, DE 19850, USA 2014.

67. Ziv-aflibercept (Zaltrap) [package insert]. Sanofi-aventis U.S. LLC, Bridgewater, NJ 08807, USA. 2012.

68. Cabozantinib (Cambometyx) [package insert]. Exelixis, Inc. South San Francisco, CA 94080 USA. 2016.

69. Mak IT, Kramer JH, Chmielinska JJ et al. EGFR-TKI, erlotinib, causes hypomagnesemia, oxidative stress, and cardiac dysfunction: attenuation by NK-1 receptor blockade. J Cardiovasc Pharmacol 2015; 65: 54-61.

70. Moore MJ, Goldstein D, Hamm J et al. Erlotinib plus gemcitabine compared with gemcitabine alone in patients with advanced pancreatic cancer: a phase III trial of the National Cancer Institute of Canada Clinical Trials Group. J Clin Oncol 2007; 25: 1960-1966.

71. Erlotinib (Tarceva) [package insert]. OSI Pharmaceuticals Inc., Melville, NY 11747, USA. . 2010.

72. Cetuximab (Erbitux) [package insert]. Bristol-Myers Squibb Company, Princeton, NJ 08543 USA. 2012.

73. Tang XM, Chen H, Liu Y et al. The cardiotoxicity of cetuximab as single therapy in Chinese chemotherapy-refractory metastatic colorectal cancer patients. Medicine (Baltimore) 2017; 96: e5946.

74. Ceritinib (Zykadia) [package insert]. Novartis Pharmaceuticals Corporation, East Hanover, New Jersey 07936, USA. 2017.

75. Crizotinib (Xalkori) [package insert]. Pfizer Labs, Division of Pfizer Inc, New York, NY 10017, USA

2012.

76. Vemurafenib (Zelboraf) [package insert]. Genentech USA, Inc., A Member of the Roche Group, 1 DNA Way, South San Francisco, CA 94080, USA. 2016.

77. Johnson DB, Balko JM, Compton ML et al. Fulminant Myocarditis with Combination Immune Checkpoint Blockade. N Engl J Med 2016; 375: 1749-1755.

78. Mahmood SS, Fradely MG, Cohen JV, et al. Myocarditis in patients treated with immune checkpoint inhibitors. J Am College Cardiol 2018; In Press.

79. Ganatra S, Neilan TG. Immune Checkpoint Inhibitor Associated Myocarditis. Oncologist 2018; In Press.

80. Bortezomib [package insert]. Millennium Pharmaceuticals Inc., 40 Landsdowne Street, Cambridge, MA 02139, USA. 2014.

81. Zangari M, Fink L, Zhan F, Tricot G. Low venous thromboembolic risk with bortezomib in multiple myeloma and potential protective effect with thalidomide/lenalidomide-based therapy: review of data from phase 3 trials and studies of novel combination regimens. Clin Lymphoma Myeloma Leuk 2011; 11: 228-236.

82. Carfilzomib [package insert]. Onyx Pharmaceuticals, Inc., Thousand Oaks, CA 91320, USA. 2016.

83. Siegel D, Martin T, Nooka A et al. Integrated safety profile of single-agent carfilzomib: experience from 526 patients enrolled in 4 phase II clinical studies. Haematologica 2013; 98: 1753-1761.

84. Stewart AK, Rajkumar SV, Dimopoulos MA et al. Carfilzomib, lenalidomide, and dexamethasone for relapsed multiple myeloma. N Engl J Med 2015; 372: 142-152.

85. Everolimus [package insert]. Novartis Pharmaceuticals Corporation, East Hanover, New Jersey 07936, USA 2010.

86. Temsirolimus (Torisel) [package insert]. Wyeth Pharmaceuticals Inc, Philadelphia, PA 19101, USA. 2015.

87. Lenalidomide (Revlimid) [package insert]. Celgene Corporation, Summit, NJ 07901, USA. 2017.

88. Thalidomide [package insert]. In. Summit, NJ 07901, USA: Celgene Corporation 2014.

89. Pomalidomide [package insert]. Celgene Corporation, Summit, NJ 07901, USA. 2013.

90. Mann BS, Johnson JR, Cohen MH et al. FDA approval summary: vorinostat for treatment of advanced primary cutaneous T-cell lymphoma. Oncologist 2007; 12: 1247-1252.

91. Vorinostat (Zolinza) [package insert]. MERCK & CO., INC. Whitehouse Station, NJ 08889, USA. 2006.

92. Belinostat (Beleodaq) [package insert]. Spectrum Pharmaceuticals, Inc., Irvine, CA 92618, USA. 2014.

93. Tamoxifen [package insert]. In. Wilmington, Delaware 19850, USA: AstraZeneca Pharmaceuticals LP 2004.

94. Toremifene [package insert]. In. Mephis, TN 38103, USA: GTx, Inc. 2011.

95. Anastrozole (Arimidex) [package insert]. In. Wilmington, DE 19850, USA: AstraZeneca Pharmaceuticals LP 2009.

96. Letrozole (femara) [package insert]. In. East Hanover, New Jersey, 07936, USA: Novartis Pharmaceuticals Corporation 2014.

97. Exemestane (Aromasin) [package insert]. In. NY 10017, USA: Pharmacia & Upjohn Co., Division of Pfizer Inc, NY 2011.

98. Goserelin (Zoladex) [package insert]. In. Wilmington, DE 19850, USA: AstraZeneca Pharmaceuticals LP 2015.

99. Leuprolide (Lupron) [package insert]. In. North Chicago, IL 60064, USA: AbbVie Inc. 2014.

100. Flutamide [package insert]. Schering Corp., Kenilworth, NJ 07033, USA. 2001.

101. Bicalutamide [package insert]. AstraZeneca Pharmaceuticals LP, Wilmington, DE 19850, USA. 2015.

102. Nilutamide [package insert]. ANI Pharmaceuticals, Inc., Baudette, MN 56623, USA. 2015.

103. Tisagenlecleucel (Kymriah) [package insert]. Novartis Pharmaceuticals Corporation, East Hanover, New Jersey 07936, USA. 2017.

104. Axicabtagene ciloleucel (Yescarta) [package insert]. Kite Pharma, Inc., Santa Monica, CA 90404, USA. 2017.

105. Ribociclib (Kisqali) [package insert]. Novartis Pharmaceuticals Corporation, East Hanover, New Jersey 07936, USA. 2017.

106. Didagelos M, Boutis A, Diamantopoulos N et al. Bleomycin cardiotoxicity during chemotherapy for an ovarian germ cell tumor. Hippokratia 2013; 17: 187-188.

107. Vogelzang NJ, Frenning DH, Kennedy BJ. Coronary artery disease after treatment with bleomycin and vinblastine. Cancer Treat Rep 1980; 64: 1159-1160.

108. Bleomycin (Blenoxane) [package insert]. Mead Johnson Oncology Products, A Bristol-Myers Subsid Co., Princeton, NJ 08543, USA.

109. Tretinoin [package insert]. Roche Laboratories Inc., 340 Kingsland Street, Nutley, NJ 07110, USA. 2004.

110. Simbre IV, Adams MJ, Deshpande SS et al. Cardiomyopathy Caused by Antineoplastic Therapies. Curr Treat Options Cardiovasc Med 2001; 3: 493-505.

111. Arsenic trioxide (Trisenox) [package insert]. Teva Pharmaceuticals USA, Inc., North Wales, PA 19454, USA. 2015.

112. Kathirgamanathan K, Angaran P, Lazo-Langner A, Gula LJ. Cardiac conduction block at multiple levels caused by arsenic trioxide therapy. Can J Cardiol 2013; 29: 130 e135-136.

Supplementary Table B: Potentially cardiotoxic cancer medicines added for the Australian context [1]

| **Medication** | **Cancer use** | **Type of toxicity** | **Frequency** |
| --- | --- | --- | --- |
| Abiraterone | Prostate | HF  Angina  Arrhythmia - atrial fibrillation  QT prolongation and Torsade de Pointes  HTN | Uncommon  Common  Common  Very rare  Very common |
| Acalabrutinib | Lymphoma | Atrial fibrillation | Common |
| Alectinib | Lung | Bradycardia  Hyperglycaemia | Very common  Very common |
| Atezolizumab | Lung, urothelial, breast, hepatocellular | Myocarditis  Hypotension  HTN  Diabetes | Rare  Common with monotherapy  Very common in combination regimens  Uncommon |
| Avelumab | Merkel cell, urothelial, renal cell | Myocarditis  MI  HF  HTN or hypotension  Diabetes | Uncommon  Common, with axitinib  Common, with axitinib  Common  Uncommon |
| Azacitidine | MDS, leukaemia | HTN and hypotension | Common |
| Bendamustine | Lymphoma, leukaemia | HF  MI  Arrhythmia- tachycardia  Vasculitis  HTN or Hypotension  Hyperglycaemia | Not known  Uncommon  Common  Uncommon  Common  Common |
| Binimetinib | Melanoma | LVD and HF  HTN  VTE | Common  Very common  Common |
| Busulfan | SCT, leukaemia  polycythaemia, myelofibrosis | LVD  Arrhythmia  Pericardial effusion/tamponade/pericarditis  HTN or hypotension  Hyperglycaemia  Thrombosis  ATE | Common  Common  Common (in 2%) in children with thalassaemia  Very common  Very common  Very common  Uncommon |
| Cabazitaxel | Prostate | Cardiac arrhythmia  HTN or hypotension  VTE  Hyperglycaemia | Common all grades, uncommon grade 3 or 4  Common all grades, uncommon grade 3 or 4  Common  Common |
| Carmustine | Glioma, myeloma, lymphoma, leukaemia | Arrhythmia- tachycardia  Hypotension | Uncommon  Uncommon |
| Cladribine | Leukaemia, lymphoma | HF  MI  Arrhythmia  Hypotension | Rare  Rare  Common tachycardia,  Uncommon atrial fibrillation  Common |
| Cobimetinib | Melanoma | LVD  HT  Hyperglycaemia | Common  Very common  Common |
| Cytarabine | Leukaemia, lymphoma | HF reported in experimental high dose therapy  Pericarditis  Bradycardia | Not known  Not known  Not known |
| Daratumumab | Multiple myeloma | Atrial fibrillation  HTN | Common in combination regimens  Very common |
| Degarelix | Prostate | QT prolongation  HTN | Uncommon  Uncommon |
| Durvalumab | Lung | Myocarditis  Diabetes | Rare  Rare |
| Encorafenib | Melanoma, colorectal | LVD  Arrhythmias  HTN  VTE | Common  Common all grade, uncommon grade 3 or 4  Common  Common |
| Entrectinib | Lung, solid tumours with NTRK gene fusion | HF  QT prolongation  Myocarditis  Hypotension  VTE- PE  Hyperglycaemia | Common  Common  Uncommon  Very common  Common  Common |
| Enzalutamide | Prostate | Ischaemic heart disease  HTN  Hyperglycaemia | Common  Very common  Very common, all grades |
| Etoposide | Lung, testicular, lymphoma, leukaemia | HF  MI  HTN or hypotension during infusion | Not known  Not known  Not known |
| Gemcitabine | Lung, pancreas, bladder, ovarian, breast, other | HF  MI  Arrhythmia  Hypotension | Rare  Rare  Rare  Rare |
| Idarubicin | Leukaemia | LVD and HF  Arrhythmia  Ischaemia  VTE  Pericarditis/myocarditis | Common (5%) at cumulative dose 150-290 mg/m^2^  Common  Not known  Common  Common |
| Lanreotide | Neuroendocrine | Sinus bradycardia  Hyperglycemia | Common  Common |
| Lenvatinib | Thyroid, renal, hepatic, endometrial | LVD  HF  MI  QT prolongation  HTN  Hypotension  ATE  Hyperlipidaemia | Common  Uncommon  Common  Common  Very common  Common  Common  Common |
| Liposomal Doxorubicin | Ovarian, breast sarcoma | LVD and HF  Arrhythmia  VTE | Lower incidence than other anthracyclines  Not known  Uncommon |
| Lorlatinib | Lung | PR interval prolongation and AV block events  Hyperlipidaemia  HTN  Hyperglycaemia | Uncommon  Very common  Very common  Common |
| Medroxyprogesterone | Prostate, endometrial, breast | MI  HF  Tachycardia  Hyperglycaemia  VTE  ATE | Not known  Not known  Not known  Not known  Not known  Not known |
| Midostaurin | Leukaemia,  systemic mastocytosis | QT prolongation  Sinus tachycardia  Pericardial effusion  HTN and hypotension | Very common  Common in AML  Common all grade, uncommon grade 3 or 4 in AML  Common |
| Mitozantrone | Breast, lymphoma, leukaemia | LVD and HF  Arrhythmia | Not known  Not known |
| Nab-paclitaxel | Pancreas, breast, lung | Cardiac arrest  LVD and HF  Arrhythmia  Hypotension or HTN  VTE PE  Hyperglycaemia | Rare  Uncommon  Common  Common  Common  Uncommon |
| Obinutuzumab | Lymphoma, leukaemia | Arrhythmia, ACS, MI, LVD (infusion reaction)  Atrial fibrillation  HTN | Not known  Common all grade, grade 3 or 4 uncommon  Common |
| Osimertinib | Lung | LVD and HF  QT prolongation | Common  Uncommon QTC >500msec |
| Panitumumab | Colorectal | Tachycardia  HTN or Hypotension  VTE | Common  Common  Common |
| Ruxolitinib | Myelofibrosis, polycythemia, essential thrombocythemia, graft vs host disease | HTN  Hyperlipidaemia | Very common  Very common |
| Thiotepa | Leukaemia SCT | LVD and HF  Arrhythmias  Myocarditis  HTN | Common (5.3%) in SCT  Very common  Uncommon  Very common |
| Trametinib | Melanoma, lung, Anaplastic thyroid cancer | HF  LVD  Bradyarrhythmia  HTN or hypotension  Hyperglycaemia  VTE | Uncommon  Common  Common  Very common  Very common with Dabrafenib  Common |
| Trastuzumab emtansine | Breast | LVD  HTN | Common all grade, grade 3 or 4 uncommon  Common |
| Trifluridine/Tipiracil | Colorectal, gastric | Ischaemia  Arrhythmia  HTN and hypotension  VTE  Prolonged QT | Uncommon  Uncommon  Uncommon  Uncommon  Uncommon |
| Triptorelin | Prostate | SCD  MI  QT prolongation  HTN  Hypotension  Hyperlipidaemia  Diabetes | Not known  Not known  Not known  Common  Rare  Uncommon  Uncommon |
| Vincristine | Leukaemia, lymphoma, sarcoma, breast, lung, others | MI  HTN  Hypotension | Not known  Not known  Not known |
| Vinorelbine | Breast, lung | HF  Ischaemia MI  Arrhythmia  HTN or hypotension | Very rare  Rare  Very rare  Uncommon |

**The frequency of toxicity was graded as:** very common (≥10%), common (≥1% to <10%), uncommon (≥0.1% to <1%), rare (≥0.01% to <0.1%), and very rare (<0.01%). Not known = Not known/incidence unable to be determined from Product Information document.

ACS, acute coronary syndrome; AI, aromatase inhibitor; ATE, arterial thromboembolism; CAD, coronary artery disease; CV, cardiovascular; CVA, cerebrovascular accident; ER, oestrogen receptor; GIST, gastrointestinal stromal tumour; HER2, human epidermal growth factor receptor 2; HF, heart failure; HTN, hypertension; IMiD, immunomodulatory drug; LHRH, luteinizing hormone-releasing hormone; LVD, left ventricular dysfunction; MCL, mantle cell lymphoma; MDS, myelodysplastic syndrome; MI, myocardial infarction; MM, multiple myeloma; mTOR, mammalian target of rapamycin; PNET, pancreatic neuroendocrine tumour; QT, corrected QT interval (preferably by Fridericia’s formula); SCC, squamous cell cancer; SCD, sudden cardiac death; SCT, stem cell transplant; TKI, tyrosine kinase inhibitor; VTE, venous thromboembolism.

**References for Supplementary Table B**

1. Therapeutic Goods Administration. Product and Consumer Medicine Information Search Facility [Website]. Australia: Australian Government Department of Health and Aged Care; [Accessed on 25/02/2023]; Available from: <https://www.ebs.tga.gov.au/ebs/picmi/picmirepository.nsf/PICMI?OpenForm&t=pi&q=>.

Supplementary Table C. Age-standardised prevalence rates per 10,000 (95% confidence interval) for the number of people alive during each calendar year with previous exposure to all potentially cardiotoxic cancer medicines and those with an expected prevalence of >1%.

|  | All persons | | Females | | Males | |
| --- | --- | --- | --- | --- | --- | --- |
| Year | All medicines | Medicines with common toxicity | All medicines | Medicines with common toxicity | All medicines | Medicines with common toxicity |
| 2005 | 49 (48·7 - 49·3) | 43 (42·7 - 43·3) | 61 (60·5 - 61·5) | 57 (56·6 - 57·4) | 39 (38·6 - 39·4) | 29 (28·7 - 29·3) |
| 2006 | 65 (64·7 - 65·3) | 57 (56·7 - 57·3) | 78 (77·5 - 78·5) | 72 (71·5 - 72·5) | 54 (53·5 - 54·5) | 42 (41·6 - 42·4) |
| 2007 | 77 (76·6 - 77·4) | 67 (66·7 - 67·3) | 91 (90·4 - 91·6) | 84 (83·5 - 84·5) | 65 (64·5 - 65·5) | 51 (50·6 - 51·4) |
| 2008 | 89 (88·6 - 89·4) | 78 (77·6 - 78·4) | 104 (103·4 - 104·6) | 96 (95·4 - 96·6) | 76 (75·5 - 76·5) | 61 (60·5 - 61·5) |
| 2009 | 99 (98·6 - 99·4) | 88 (87·6 - 88·4) | 116 (115·4 - 116·6) | 107 (106·4 - 107·6) | 85 (84·4 - 85·6) | 69 (68·5 - 69·5) |
| 2010 | 109 (108·6 - 109·4) | 96 (95·6 - 96·4) | 127 (126·4 - 127·6) | 118 (117·4 - 118·6) | 93 (92·4 - 93·6) | 76 (75·5 - 76·5) |
| 2011 | 118 (117·6 - 118·4) | 105 (104·6 - 105·4) | 138 (137·3 - 138·7) | 128 (127·4 - 128·6) | 101 (100·4 - 101·6) | 83 (82·5 - 83·5) |
| 2012 | 129 (128·6 - 129·4) | 115 (114·6 - 115·4) | 150 (149·3 - 150·7) | 139 (138·4 - 139·6) | 110 (109·4 - 110·6) | 91 (90·4 - 91·6) |
| 2013 | 138 (137·5 - 138·5) | 123 (122·6 - 123·4) | 162 (161·3 - 162·7) | 151 (150·3 - 151·7) | 116 (115·4 - 116·6) | 97 (96·4 - 97·6) |
| 2014 | 148 (147·5 - 148·5) | 133 (132·6 - 133·4) | 175 (174·3 - 175·7) | 163 (162·3 - 163·7) | 123 (122·4 - 123·6) | 104 (103·4 - 104·6) |
| 2015 | 159 (158·5 - 159·5) | 143 (142·5 - 143·5) | 189 (188·3 - 189·7) | 176 (175·3 - 176·7) | 131 (130·4 - 131·6) | 111 (110·4 - 111·6) |
| 2016 | 170 (169·5 - 170·5) | 153 (152·5 - 153·5) | 204 (203·2 - 204·8) | 190 (189·3 - 190·7) | 137 (136·4 - 137·6) | 117 (116·4 - 117·6) |
| 2017 | 181 (180·5 - 181·5) | 164 (163·5 - 164·5) | 219 (218·2 - 219·8) | 205 (204·2 - 205·8) | 144 (143·4 - 144·6) | 123 (122·4 - 123·6) |
| 2018 | 194 (193·5 - 194·5) | 177 (176·5 - 177·5) | 237 (236·2 - 237·8) | 223 (222·2 - 223·8) | 150 (149·3 - 150·7) | 129 (128·4 - 129·6) |
| 2019 | 206 (205·5 - 206·5) | 189 (188·5 - 189·5) | 255 (254·2 - 255·8) | 240 (239·2 - 240·8) | 157 (156·3 - 157·7) | 136 (135·4 - 136·6) |
| 2020 | 218 (217·5 - 218·5) | 200 (199·5 - 200·5) | 272 (271·2 - 272·8) | 257 (256·2 - 257·8) | 163 (162·3 - 163·7) | 141 (140·4 - 141·6) |
| 2021 | 232 (231·4 - 232·6) | 213 (212·5 - 213·5) | 293 (292·1 - 293·9) | 278 (277·1 - 278·9) | 169 (168·3 - 169·7) | 146 (145·4 - 146·6) |

Supplementary Table D. Age-specific prevalence rates per 10,000 (95% confidence interval) of the number of people exposed to potentially cardiotoxic cancer medicines and alive during each calendar.

|  | Age groups |  |  |  |  |  |  |  |  |
| --- | --- | --- | --- | --- | --- | --- | --- | --- | --- |
| Year | 0 – 9 | 10 – 19 | 20 – 29 | 30 – 39 | 40 – 49 | 50 – 59 | 60 – 69 | 70 – 74 | 75+ |
| 2005 | 0 (0·2 - 0·3) | 1 (0·7 - 0·9) | 4 (3·7 - 4·2) | 11 (10·8 - 11·6) | 34 (32·8 - 34·2) | 79 (77·6 - 79·7) | 159 (157 - 160·8) | 216 (212·8 - 220·1) | 233 (230·2 - 235·6) |
| 2006 | 0 (0·4 - 0·5) | 2 (1·4 - 1·7) | 7 (6·8 - 7·4) | 16 (15·9 - 16·8) | 45 (44 - 45·5) | 102 (100·5 - 103) | 207 (204·6 - 208·9) | 287 (282·6 - 291) | 300 (297·4 - 303·5) |
| 2007 | 1 (0·6 - 0·8) | 2 (1·9 - 2·2) | 9 (8·9 - 9·6) | 20 (19·8 - 20·9) | 54 (53·1 - 54·8) | 119 (117·7 - 120·4) | 242 (239·7 - 244·1) | 350 (345·3 - 354·4) | 352 (349·2 - 355·6) |
| 2008 | 1 (0·8 - 1·1) | 3 (2·3 - 2·7) | 11 (10·8 - 11·6) | 25 (24·7 - 25·8) | 61 (60·5 - 62·2) | 136 (134·7 - 137·5) | 268 (266 - 270·6) | 406 (401·6 - 411·3) | 408 (404·2 - 411·1) |
| 2009 | 1 (0·8 - 1·1) | 3 (2·7 - 3·1) | 13 (12·4 - 13·2) | 29 (28·3 - 29·5) | 69 (67·7 - 69·5) | 154 (152·1 - 155) | 294 (291·3 - 296) | 454 (449·3 - 459·4) | 458 (454·1 - 461·3) |
| 2010 | 1 (1 - 1·2) | 3 (3·1 - 3·5) | 14 (13·9 - 14·8) | 33 (32·8 - 34·1) | 75 (73·7 - 75·7) | 167 (165·6 - 168·7) | 317 (314·1 - 318·9) | 492 (487 - 497·3) | 513 (508·9 - 516·5) |
| 2011 | 1 (1·1 - 1·4) | 3 (3·2 - 3·7) | 16 (15·2 - 16·1) | 38 (37·4 - 38·8) | 84 (82·5 - 84·6) | 179 (177·9 - 181) | 340 (337·3 - 342·2) | 526 (520·4 - 530·9) | 564 (560·4 - 568·3) |
| 2012 | 2 (1·5 - 1·8) | 4 (3·7 - 4·2) | 18 (17·7 - 18·6) | 42 (41·2 - 42·6) | 90 (88·8 - 90·8) | 196 (194 - 197·3) | 364 (361·5 - 366·5) | 556 (550·4 - 561·1) | 627 (622·8 - 630·9) |
| 2013 | 2 (1·9 - 2·2) | 4 (4·1 - 4·6) | 19 (18·9 - 19·8) | 47 (46·5 - 48) | 97 (95·4 - 97·6) | 208 (205·9 - 209·2) | 384 (381·6 - 386·6) | 601 (595·5 - 606·4) | 678 (674 - 682·4) |
| 2014 | 2 (2·2 - 2·5) | 5 (4·8 - 5·3) | 21 (20·3 - 21·3) | 53 (52·3 - 53·9) | 105 (103·8 - 106) | 222 (220·5 - 223·9) | 406 (403·9 - 409) | 627 (621·6 - 632·5) | 736 (731·6 - 740·3) |
| 2015 | 4 (3·3 - 3·7) | 6 (6·1 - 6·7) | 24 (23·9 - 25) | 58 (57·6 - 59·3) | 115 (114·1 - 116·4) | 235 (233·6 - 237·1) | 432 (429·7 - 434·9) | 653 (647·2 - 658) | 793 (788·3 - 797·2) |
| 2016 | 4 (3·6 - 4) | 7 (7·2 - 7·8) | 29 (28·2 - 29·3) | 70 (69·1 - 70·9) | 126 (124·7 - 127·1) | 249 (247·5 - 251·1) | 443 (440·8 - 446) | 691 (685·2 - 696·1) | 847 (842·2 - 851·2) |
| 2017 | 4 (4·1 - 4·6) | 8 (8·1 - 8·8) | 32 (31·1 - 32·2) | 84 (83·5 - 85·4) | 137 (135·3 - 137·9) | 258 (256 - 259·6) | 465 (462·1 - 467·4) | 721 (715·6 - 726·4) | 900 (895·5 - 904·7) |
| 2018 | 5 (4·4 - 4·9) | 10 (9·4 - 10·1) | 35 (34·6 - 35·9) | 104 (103·4 - 105·6) | 149 (147·7 - 150·4) | 269 (267 - 270·6) | 487 (483·9 - 489·3) | 731 (725·6 - 736·1) | 961 (956·7 - 966·1) |
| 2019 | 5 (4·7 - 5·1) | 11 (10·3 - 11·1) | 38 (37·8 - 39) | 125 (123·9 - 126·2) | 163 (161·6 - 164·3) | 280 (278·5 - 282·3) | 504 (500·9 - 506·4) | 768 (762·8 - 773·4) | 1,012 (1,007·8 - 1,017·2) |
| 2020 | 4 (4·2 - 4·6) | 12 (11·8 - 12·6) | 43 (42 - 43·3) | 146 (145·1 - 147·5) | 178 (176·5 - 179·3) | 289 (287 - 290·7) | 513 (510·4 - 515·8) | 800 (795·1 - 805·6) | 1,056 (1,051·7 - 1,061·2) |
| 2021 | 5 (4·5 - 5) | 13 (12·8 - 13·6) | 49 (48·3 - 49·8) | 169 (168 - 170·6) | 198 (196·6 - 199·7) | 301 (298·9 - 302·8) | 531 (528·7 - 534·2) | 811 (805·7 - 816·1) | 1,113 (1,108 - 1,117·5) |

Supplementary Table E. Age-standardised prevalence rates per 10,000 (95% confidence interval) of the number of people exposed to potentially cardiotoxic cancer medicines and alive during each calendar year after five and 10 years from first exposure. Overall and by sex.

|  | Overall | | Females | | Males | |
| --- | --- | --- | --- | --- | --- | --- |
| Year | Five-year prevalence | Ten-year prevalence | Five-year prevalence | Ten-year prevalence | Five-year prevalence | Ten-year prevalence |
| 2005 | - | - | - | - | - | - |
| 2006 | - | - | - | - | - | - |
| 2007 | - | - | - | - | - | - |
| 2008 | - | - | - | - | - | - |
| 2009 | 29 (28·8 - 29·2) | - | 40 (39·6 - 40·4) | - | 18 (17·7 - 18·3) | - |
| 2010 | 38 (37·8 - 38·2) | - | 51 (50·6 - 51·4) | - | 26 (25·7 - 26·3) | - |
| 2011 | 47 (46·7 - 47·3) | - | 61 (60·6 - 61·4) | - | 33 (32·7 - 33·3) | - |
| 2012 | 55 (54·7 - 55·3) | - | 71 (70·5 - 71·5) | - | 40 (39·6 - 40·4) | - |
| 2013 | 64 (63·7 - 64·3) | - | 81 (80·5 - 81·5) | - | 46 (45·6 - 46·4) | - |
| 2014 | 71 (70·7 - 71·3) | 22 (21·8 - 22·2) | 90 (89·5 - 90·5) | 33 (32·7 - 33·3) | 52 (51·6 - 52·4) | 11 (10·8 - 11·2) |
| 2015 | 79 (78·7 - 79·3) | 31 (30·8 - 31·2) | 100 (99·5 - 100·5) | 44 (43·6 - 44·4) | 58 (57·6 - 58·4) | 18 (17·7 - 18·3) |
| 2016 | 87 (86·6 - 87·4) | 39 (38·7 - 39·3) | 110 (109·4 - 110·6) | 53 (52·6 - 53·4) | 63 (62·6 - 63·4) | 24 (23·7 - 24·3) |
| 2017 | 94 (93·6 - 94·4) | 46 (45·7 - 46·3) | 120 (119·4 - 120·6) | 62 (61·6 - 62·4) | 68 (67·6 - 68·4) | 29 (28·7 - 29·3) |
| 2018 | 102 (101·6 - 102·4) | 53 (52·7 - 53·3) | 130 (129·4 - 130·6) | 71 (70·5 - 71·5) | 73 (72·5 - 73·5) | 34 (33·7 - 34·3) |
| 2019 | 110 (109·6 - 110·4) | 59 (58·7 - 59·3) | 141 (140·4 - 141·6) | 80 (79·5 - 80·5) | 78 (77·5 - 78·5) | 38 (37·7 - 38·3) |
| 2020 | 119 (118·6 - 119·4) | 66 (65·7 - 66·3) | 154 (153·4 - 154·6) | 88 (87·5 - 88·5) | 84 (83·5 - 84·5) | 43 (42·6 - 43·4) |
| 2021 | 134 (133·6 - 134·4) | 76 (75·7 - 76·3) | 172 (171·3 - 172·7) | 99 (98·5 - 99·5) | 95 (94·5 - 95·5) | 51 (50·6 - 51·4) |

Supplementary Table F. Age-standardised prevalence rates per 10,000 (95% confidence interval) of the number of people exposed to potentially cardiotoxic cancer medicines and alive during each calendar year, exposed to N medicines (i.e., 1, 2, or 3 or more). Overall and by sex.

|  | Overall | | | Females | | | Males | | |
| --- | --- | --- | --- | --- | --- | --- | --- | --- | --- |
| Year | One medicine | Two medicines | 3 or more medicines | One medicine | Two medicines | 3 or more medicines | One medicine | Two medicines | 3 or more medicines |
| 2005 | 39 (38·7 - 39·3) | 7 (6·9 - 7·1) | 3 (2·9 - 3·1) | 57 (56·5 - 57·5) | 8 (7·8 - 8·2) | 4 (3·9 - 4·1) | 30 (29·7 - 30·3) | 7 (6·8 - 7·2) | 2 (1·9 - 2·1) |
| 2006 | 46 (45·7 - 46·3) | 12 (11·9 - 12·1) | 7 (6·9 - 7·1) | 55 (54·6 - 55·4) | 13 (12·8 - 13·2) | 10 (9·8 - 10·2) | 39 (38·6 - 39·4) | 11 (10·8 - 11·2) | 4 (3·9 - 4·1) |
| 2007 | 52 (51·7 - 52·3) | 15 (14·8 - 15·2) | 10 (9·9 - 10·1) | 59 (58·6 - 59·4) | 17 (16·8 - 17·2) | 15 (14·8 - 15·2) | 47 (46·6 - 47·4) | 13 (12·8 - 13·2) | 5 (4·9 - 5·1) |
| 2008 | 57 (56·7 - 57·3) | 17 (16·8 - 17·2) | 14 (13·8 - 14·2) | 63 (62·5 - 63·5) | 20 (19·7 - 20·3) | 20 (19·7 - 20·3) | 53 (52·6 - 53·4) | 15 (14·8 - 15·2) | 8 (7·8 - 8·2) |
| 2009 | 62 (61·7 - 62·3) | 20 (19·8 - 20·2) | 17 (16·8 - 17·2) | 68 (67·5 - 68·5) | 22 (21·7 - 22·3) | 25 (24·7 - 25·3) | 57 (56·5 - 57·5) | 18 (17·7 - 18·3) | 9 (8·8 - 9·2) |
| 2010 | 66 (65·7 - 66·3) | 22 (21·8 - 22·2) | 20 (19·8 - 20·2) | 72 (71·5 - 72·5) | 25 (24·7 - 25·3) | 30 (29·7 - 30·3) | 62 (61·5 - 62·5) | 20 (19·7 - 20·3) | 11 (10·8 - 11·2) |
| 2011 | 70 (69·7 - 70·3) | 24 (23·8 - 24·2) | 24 (23·8 - 24·2) | 76 (75·5 - 76·5) | 27 (26·7 - 27·3) | 35 (34·7 - 35·3) | 66 (65·5 - 66·5) | 22 (21·7 - 22·3) | 13 (12·8 - 13·2) |
| 2012 | 75 (74·7 - 75·3) | 26 (25·8 - 26·2) | 28 (27·8 - 28·2) | 82 (81·5 - 82·5) | 28 (27·7 - 28·3) | 40 (39·6 - 40·4) | 70 (69·5 - 70·5) | 24 (23·7 - 24·3) | 16 (15·8 - 16·2) |
| 2013 | 79 (78·7 - 79·3) | 28 (27·8 - 28·2) | 31 (30·8 - 31·2) | 87 (86·5 - 87·5) | 30 (29·7 - 30·3) | 45 (44·6 - 45·4) | 73 (72·5 - 73·5) | 26 (25·7 - 26·3) | 17 (16·8 - 17·2) |
| 2014 | 84 (83·6 - 84·4) | 29 (28·8 - 29·2) | 35 (34·8 - 35·2) | 93 (92·5 - 93·5) | 31 (30·7 - 31·3) | 50 (49·6 - 50·4) | 76 (75·5 - 76·5) | 27 (26·7 - 27·3) | 20 (19·8 - 20·2) |
| 2015 | 88 (87·6 - 88·4) | 31 (30·8 - 31·2) | 39 (38·8 - 39·2) | 100 (99·5 - 100·5) | 33 (32·7 - 33·3) | 56 (55·6 - 56·4) | 78 (77·5 - 78·5) | 30 (29·7 - 30·3) | 23 (22·7 - 23·3) |
| 2016 | 94 (93·6 - 94·4) | 33 (32·8 - 33·2) | 43 (42·8 - 43·2) | 108 (107·4 - 108·6) | 35 (34·7 - 35·3) | 61 (60·6 - 61·4) | 81 (80·5 - 81·5) | 31 (30·7 - 31·3) | 25 (24·7 - 25·3) |
| 2017 | 100 (99·6 - 100·4) | 35 (34·8 - 35·2) | 46 (45·7 - 46·3) | 117 (116·4 - 117·6) | 37 (36·7 - 37·3) | 65 (64·6 - 65·4) | 83 (82·5 - 83·5) | 33 (32·7 - 33·3) | 27 (26·7 - 27·3) |
| 2018 | 107 (106·6 - 107·4) | 37 (36·8 - 37·2) | 50 (49·7 - 50·3) | 128 (127·4 - 128·6) | 39 (38·7 - 39·3) | 70 (69·6 - 70·4) | 86 (85·5 - 86·5) | 34 (33·7 - 34·3) | 30 (29·7 - 30·3) |
| 2019 | 115 (114·6 - 115·4) | 39 (38·8 - 39·2) | 53 (52·7 - 53·3) | 140 (139·4 - 140·6) | 41 (40·7 - 41·3) | 74 (73·6 - 74·4) | 89 (88·5 - 89·5) | 37 (36·7 - 37·3) | 31 (30·7 - 31·3) |
| 2020 | 122 (121·6 - 122·4) | 40 (39·8 - 40·2) | 55 (54·7 - 55·3) | 152 (151·4 - 152·6) | 42 (41·7 - 42·3) | 77 (76·6 - 77·4) | 92 (91·5 - 92·5) | 38 (37·7 - 38·3) | 33 (32·7 - 33·3) |
| 2021 | 131 (130·6 - 131·4) | 42 (41·8 - 42·2) | 58 (57·7 - 58·3) | 168 (167·3 - 168·7) | 45 (44·7 - 45·3) | 81 (80·5 - 81·5) | 94 (93·5 - 94·5) | 41 (40·7 - 41·3) | 34 (33·7 - 34·3) |

Supplementary Table G. Age-standardised prevalence rates per 10,000 (95% confidence interval) of the number of people exposed to potentially cardiotoxic cancer medicines and alive during each calendar year after N years (ie, 1, 5, and 10 years), exposed to N medicines (i.e., 1, 2, or 3 or more).

|  | One medicine | | Two medicines | | Three or more medicines | |
| --- | --- | --- | --- | --- | --- | --- |
| Year | Five-year prevalence | Ten-year prevalence | Five-year prevalence | Ten-year prevalence | Five-year prevalence | Ten-year prevalence |
| 2005 | - | - | - | - | - | - |
| 2006 | - | - | - | - | - | - |
| 2007 | - | - | - | - | - | - |
| 2008 | - | - | - | - | - | - |
| 2009 | 19 (18·7 - 19·3) | - | 7 (6·8 - 7·2) | - | 4 (3·8 - 4·2) | - |
| 2010 | 24 (23·7 - 24·3) | - | 9 (8·8 - 9·2) | - | 7 (6·8 - 7·2) | - |
| 2011 | 29 (28·7 - 29·3) | - | 11 (10·8 - 11·2) | - | 10 (9·8 - 10·2) | - |
| 2012 | 34 (33·7 - 34·3) | - | 12 (11·8 - 12·2) | - | 12 (11·8 - 12·2) | - |
| 2013 | 38 (37·7 - 38·3) | - | 14 (13·8 - 14·2) | - | 15 (14·8 - 15·2) | - |
| 2014 | 42 (41·6 - 42·4) | 14 (13·6 - 14·4) | 15 (14·8 - 15·2) | 6 (5·8 - 6·2) | 20 (19·8 - 20·2) | 4 (3·8 - 4·2) |
| 2015 | 46 (45·6 - 46·4) | 19 (18·6 - 19·4) | 16 (15·8 - 16·2) | 7 (6·8 - 7·2) | 21 (20·8 - 21·2) | 6 (5·8 - 6·2) |
| 2016 | 49 (48·6 - 49·4) | 23 (22·6 - 23·4) | 18 (17·8 - 18·2) | 9 (8·8 - 9·2) | 24 (23·8 - 24·2) | 9 (8·8 - 9·2) |
| 2017 | 53 (52·6 - 53·4) | 27 (26·6 - 27·4) | 19 (18·8 - 19·2) | 10 (9·8 - 10·2) | 27 (26·7 - 27·3) | 11 (10·7 - 11·3) |
| 2018 | 56 (55·6 - 56·4) | 30 (29·6 - 30·4) | 20 (19·8 - 20·2) | 11 (10·8 - 11·2) | 30 (29·7 - 30·3) | 14 (13·7 - 14·3) |
| 2019 | 60 (59·6 - 60·4) | 33 (32·6 - 33·4) | 21 (20·8 - 21·2) | 12 (11·8 - 12·2) | 33 (32·7 - 33·3) | 16 (15·7 - 16·3) |
| 2020 | 65 (64·6 - 65·4) | 36 (35·6 - 36·4) | 23 (22·8 - 23·2) | 13 (12·8 - 13·2) | 36 (35·7 - 36·3) | 18 (17·7 - 18·3) |
| 2021 | 71 (70·6 - 71·4) | 40 (39·6 - 40·4) | 24 (23·8 - 24·2) | 14 (13·8 - 14·2) | 38 (37·7 - 38·3) | 21 (20·7 - 21·3) |

Supplementary Table H. Age-standardised prevalence rates per 10,000 (95% confidence interval) for the number of people alive during each calendar year with previous exposure to all potentially cardiotoxic cancer medicines, excluding tamoxifen and aromatase inhibitors, and those with an expected prevalence of >1%.

|  | All persons | | Females | | Males | |
| --- | --- | --- | --- | --- | --- | --- |
| Year | All medicines | Medicines with common toxicity | All medicines | Medicines with common toxicity | All medicines | Medicines with common toxicity |
| 2005 | 29 (28·8 - 29·2) | 22 (21·8 - 22·2) | 23 (22·7 - 23·3) | 18 (17·7 - 18·3) | 38 (37·6 - 38·4) | 28 (27·7 - 28·3) |
| 2006 | 42 (41·7 - 42·3) | 33 (32·8 - 33·2) | 35 (34·6 - 35·4) | 29 (28·7 - 29·3) | 52 (51·5 - 52·5) | 40 (39·6 - 40·4) |
| 2007 | 52 (51·7 - 52·3) | 42 (41·7 - 42·3) | 46 (45·6 - 46·4) | 38 (37·6 - 38·4) | 63 (62·5 - 63·5) | 49 (48·6 - 49·4) |
| 2008 | 62 (61·7 - 62·3) | 51 (50·7 - 51·3) | 56 (55·6 - 56·4) | 47 (46·6 - 47·4) | 72 (71·5 - 72·5) | 58 (57·5 - 58·5) |
| 2009 | 71 (70·7 - 71·3) | 59 (58·7 - 59·3) | 65 (64·5 - 65·5) | 56 (55·6 - 56·4) | 81 (80·5 - 81·5) | 65 (64·5 - 65·5) |
| 2010 | 79 (78·6 - 79·4) | 66 (65·7 - 66·3) | 74 (73·5 - 74·5) | 65 (64·5 - 65·5) | 89 (88·4 - 89·6) | 72 (71·5 - 72·5) |
| 2011 | 87 (86·6 - 87·4) | 74 (73·7 - 74·3) | 83 (82·5 - 83·5) | 72 (71·5 - 72·5) | 96 (95·4 - 96·6) | 78 (77·5 - 78·5) |
| 2012 | 96 (95·6 - 96·4) | 81 (80·6 - 81·4) | 92 (91·5 - 92·5) | 81 (80·5 - 81·5) | 104 (103·4 - 104·6) | 86 (85·5 - 86·5) |
| 2013 | 103 (102·6 - 103·4) | 88 (87·6 - 88·4) | 102 (101·4 - 102·6) | 89 (88·5 - 89·5) | 110 (109·4 - 110·6) | 91 (90·5 - 91·5) |
| 2014 | 111 (110·6 - 111·4) | 95 (94·6 - 95·4) | 111 (110·4 - 111·6) | 98 (97·5 - 98·5) | 116 (115·4 - 116·6) | 96 (95·4 - 96·6) |
| 2015 | 120 (119·6 - 120·4) | 103 (102·6 - 103·4) | 121 (120·4 - 121·6) | 107 (106·4 - 107·6) | 123 (122·4 - 123·6) | 102 (101·4 - 102·6) |
| 2016 | 127 (126·6 - 127·4) | 110 (109·6 - 110·4) | 130 (129·4 - 130·6) | 115 (114·4 - 115·6) | 128 (127·4 - 128·6) | 107 (106·4 - 107·6) |
| 2017 | 134 (133·6 - 134·4) | 117 (116·6 - 117·4) | 140 (139·4 - 140·6) | 125 (124·4 - 125·6) | 133 (132·4 - 133·6) | 112 (111·4 - 112·6) |
| 2018 | 143 (142·6 - 143·4) | 125 (124·6 - 125·4) | 151 (150·4 - 151·6) | 136 (135·4 - 136·6) | 138 (137·4 - 138·6) | 117 (116·4 - 117·6) |
| 2019 | 151 (150·5 - 151·5) | 133 (132·6 - 133·4) | 161 (160·3 - 161·7) | 145 (144·4 - 145·6) | 144 (143·4 - 144·6) | 123 (122·4 - 123·6) |
| 2020 | 158 (157·5 - 158·5) | 139 (138·6 - 139·4) | 169 (168·3 - 169·7) | 153 (152·4 - 153·6) | 149 (148·4 - 149·6) | 127 (126·4 - 127·6) |
| 2021 | 166 (165·5 - 166·5) | 147 (146·6 - 147·4) | 181 (180·3 - 181·7) | 164 (163·3 - 164·7) | 155 (154·4 - 155·6) | 132 (131·4 - 132·6) |

Supplementary Table I. Age-specific prevalence rates per 10,000 (95% confidence interval) of the number of people exposed to potentially cardiotoxic cancer medicines, excluding tamoxifen and aromatase inhibitors, and alive during each calendar.

|  | Age groups |  |  |  |  |  |  |  |  |
| --- | --- | --- | --- | --- | --- | --- | --- | --- | --- |
| Year | 0 – 9 | 10 – 19 | 20 – 29 | 30 – 39 | 40 – 49 | 50 – 59 | 60 – 69 | 70 – 74 | 75+ |
| 2005 | 0 (0·2 - 0·3) | 1 (0·7 - 0·9) | 3 (3·1 - 3·5) | 8 (8 - 8·7) | 18 (17·8 - 18·7) | 39 (37·8 - 39·4) | 90 (88·6 - 91·4) | 141 (137·9 - 143·8) | 143 (140·6 - 144·8) |
| 2006 | 0 (0·3 - 0·5) | 1 (1·3 - 1·5) | 6 (6·0 - 6·5) | 13 (12·3 - 13·1) | 29 (28·2 - 29·5) | 59 (57·6 - 59·5) | 129 (127·4 - 130·7) | 197 (193·3 - 200·2) | 196 (193·6 - 198·4) |
| 2007 | 1 (0·6 - 0·8) | 2 (1·7 - 2·0) | 8 (7·8 - 8·4) | 17 (16·1 - 17·0) | 37 (36·6 - 37·9) | 73 (72·1 - 74·2) | 159 (157·0 - 160·6) | 249 (244·9 - 252·7) | 237 (234·3 - 239·6) |
| 2008 | 1 (0·8 - 1·0) | 2 (2·0 - 2·4) | 9 (9·0 - 9·7) | 21 (20·2 - 21·2) | 45 (44·4 - 45·9) | 89 (87·9 - 90·2) | 180 (178·4 - 182·2) | 292 (288·3 - 296·6) | 282 (279·2 – 285·0) |
| 2009 | 1 (0·8 - 1·0) | 2 (2·3 - 2·6) | 10 (10 - 10·7) | 24 (23·3 - 24·4) | 53 (52·1 - 53·7) | 106 (104·8 - 107·3) | 200 (197·6 - 201·5) | 329 (325·1 - 333·7) | 323 (320·4 - 326·5) |
| 2010 | 1 (1·0 - 1·2) | 3 (2·7 - 3·1) | 12 (11·3 - 12·0) | 27 (26·7 - 27·9) | 59 (58·6 - 60·3) | 119 (117·4 – 120·0) | 219 (217·4 - 221·4) | 358 (353·2 - 362·1) | 369 (365·8 - 372·2) |
| 2011 | 1 (1·1 - 1·4) | 3 (2·8 - 3·2) | 12 (12·0 - 12·8) | 31 (30·3 - 31·5) | 68 (67·3 - 69·1) | 130 (129·1 - 131·8) | 239 (236·7 - 240·8) | 384 (379·3 - 388·3) | 409 (405·4 - 412·1) |
| 2012 | 2 (1·5 - 1·8) | 3 (3·1 - 3·5) | 14 (13·8 - 14·6) | 33 (32·4 - 33·7) | 74 (73·0 - 74·9) | 145 (143·2 - 146·0) | 261 (259·2 - 263·4) | 411 (406·4 - 415·6) | 457 (453·6 - 460·6) |
| 2013 | 2 (1·8 - 2·1) | 4 (3·5 - 3·9) | 15 (14·3 - 15·1) | 36 (35·7 - 37·1) | 80 (78·7 - 80·7) | 156 (154·7 - 157·6) | 279 (276·4 - 280·7) | 442 (437·4 - 446·7) | 496 (492·1 - 499·3) |
| 2014 | 2 (2·0 - 2·4) | 4 (4·0 - 4·5) | 15 (14·9 - 15·7) | 39 (38·2 - 39·5) | 87 (86·2 - 88·2) | 170 (169·0 - 172·0) | 298 (296·0 - 300·4) | 462 (456·8 - 466·2) | 540 (536·6 - 544·1) |
| 2015 | 3 (3·2 - 3·6) | 5 (5·1 - 5·7) | 17 (16·1 - 16·9) | 40 (39·7 - 41·0) | 95 (94·3 - 96·5) | 183 (181·4 - 184·4) | 320 (317·7 - 322·1) | 482 (477·0 - 486·4) | 585 (581·4 - 589·0) |
| 2016 | 4 (3·4 - 3·9) | 6 (6·1 - 6·7) | 17 (16·4 - 17·2) | 43 (42·6 - 44·0) | 102 (101·3 - 103·6) | 195 (193·8 - 197·0) | 331 (328·3 - 332·8) | 512 (507·4 - 516·8) | 625 (621·2 - 628·9) |
| 2017 | 4 (4·0 - 4·5) | 7 (6·9 - 7·5) | 17 (16·9 - 17·8) | 49 (47·8 - 49·3) | 109 (108·1 - 110·4) | 202 (200·4 - 203·6) | 347 (345·0 - 349·6) | 540 (535·6 - 544·9) | 668 (664·0 - 671·9) |
| 2018 | 5 (4·3 - 4·8) | 9 (8·2 - 8·9) | 19 (18·2 - 19·1) | 55 (54·3 - 55·9) | 116 (115·2 - 117·6) | 212 (210·4 - 213·6) | 368 (366·1 - 370·8) | 550 (545·3 - 554·4) | 715 (711·2 - 719·3) |
| 2019 | 5 (4·6 - 5·0) | 9 (9·1 - 9·8) | 20 (19·1 - 20·0) | 61 (60·1 - 61·7) | 124 (123·0 - 125·4) | 222 (220·6 - 223·9) | 385 (383·0 - 387·8) | 581 (576·0 - 585·2) | 753 (749·1 - 757·2) |
| 2020 | 4 (4·0 - 4·5) | 11 (10·5 - 11·3) | 21 (20·7 - 21·6) | 66 (64·8 - 66·5) | 132 (130·6 - 133·0) | 231 (229·1 - 232·4) | 395 (393·0 - 397·8) | 607 (601·9 - 611·1) | 789 (785·1 - 793·3) |
| 2021 | 5 (4·3 - 4·8) | 12 (11·5 - 12·3) | 23 (22·8 - 23·9) | 71 (70·4 - 72·1) | 143 (141·5 - 144·1) | 240 (238·5 – 242·0) | 413 (410·1 - 414·9) | 619 (614·0 - 623·2) | 836 (831·7 - 839·9) |

Supplementary Table J. Age-standardised prevalence rates per 10,000 (95% confidence interval) of the number of people exposed to potentially cardiotoxic cancer medicines, excluding tamoxifen and aromatase inhibitors, and alive during each calendar year after five and 10 years from first exposure.
Overall and by sex.

|  | Overall | | Females | | Males | |
| --- | --- | --- | --- | --- | --- | --- |
| Year | Five-year prevalence | Ten-year prevalence | Five-year prevalence | Ten-year prevalence | Five-year prevalence | Ten-year prevalence |
| 2005 | - | - | - | - | - | - |
| 2006 | - | - | - | - | - | - |
| 2007 | - | - | - | - | - | - |
| 2008 | - | - | - | - | - | - |
| 2009 | 13 (12·8 - 13·2) | - | 11 (10·8 - 11·2) | - | 17 (16·7 - 17·3) | - |
| 2010 | 21 (20·8 - 21·2) | - | 19 (18·7 - 19·3) | - | 24 (23·7 - 24·3) | - |
| 2011 | 28 (27·8 - 28·2) | - | 27 (26·7 - 27·3) | - | 31 (30·7 - 31·3) | - |
| 2012 | 35 (34·8 - 35·2) | - | 35 (34·7 - 35·3) | - | 37 (36·6 - 37·4) | - |
| 2013 | 42 (41·7 - 42·3) | - | 43 (42·6 - 43·4) | - | 43 (42·6 - 43·4) | - |
| 2014 | 48 (47·7 - 48·3) | 10 (9·9 - 10·1) | 50 (49·6 - 50·4) | 10 (9·8 - 10·2) | 49 (48·6 - 49·4) | 10 (9·8 - 10·2) |
| 2015 | 54 (53·7 - 54·3) | 16 (15·8 - 16·2) | 58 (57·6 - 58·4) | 17 (16·8 - 17·2) | 53 (52·6 - 53·4) | 16 (15·8 - 16·2) |
| 2016 | 61 (60·7 - 61·3) | 23 (22·8 - 23·2) | 65 (64·6 - 65·4) | 24 (23·7 - 24·3) | 59 (58·6 - 59·4) | 22 (21·7 - 22·3) |
| 2017 | 67 (66·7 - 67·3) | 28 (27·8 - 28·2) | 73 (72·5 - 73·5) | 31 (30·7 - 31·3) | 63 (62·6 - 63·4) | 26 (25·7 - 26·3) |
| 2018 | 73 (72·7 - 73·3) | 34 (33·8 - 34·2) | 80 (79·5 - 80·5) | 38 (37·7 - 38·3) | 67 (66·6 - 67·4) | 31 (30·7 - 31·3) |
| 2019 | 78 (77·7 - 78·3) | 39 (38·8 - 39·2) | 88 (87·5 - 88·5) | 45 (44·6 - 45·4) | 71 (70·6 - 71·4) | 34 (33·7 - 34·3) |
| 2020 | 85 (84·7 - 85·3) | 45 (44·7 - 45·3) | 95 (94·5 - 95·5) | 52 (51·6 - 52·4) | 76 (75·6 - 76·4) | 39 (38·7 - 39·3) |
| 2021 | 95 (94·7 - 95·3) | 52 (51·7 - 52·3) | 106 (105·5 - 106·5) | 60 (59·6 - 60·4) | 85 (84·5 - 85·5) | 46 (45·6 - 46·4) |

Supplementary Table K. Age-standardised prevalence rates per 10,000 (95% confidence interval) of the number of people exposed to potentially cardiotoxic cancer medicines, excluding tamoxifen and aromatase inhibitors, and alive during each calendar year, exposed to N medicines (i.e., 1, 2, or 3 or more). Overall and by sex.

|  | Overall | | | Females | | | Males | | |
| --- | --- | --- | --- | --- | --- | --- | --- | --- | --- |
| Year | One medicine | Two medicines | 3 or more medicines | One medicine | Two medicines | 3 or more medicines | One medicine | Two medicines | 3 or more medicines |
| 2005 | 21 (20·8 - 21·2) | 6 (5·9 - 6·1) | 2 (1·9 - 2·1) | 18 (17·7 - 18·3) | 5 (4·9 - 5·1) | 3 (2·9 - 3·1) | 29 (28·7 - 29·3) | 7 (6·8 - 7·2) | 2 (1·9 - 2·1) |
| 2006 | 28 (27·8 - 28·2) | 9 (8·9 - 9·1) | 5 (4·9 - 5·1) | 21 (20·7 - 21·3) | 8 (7·8 - 8·2) | 6 (5·9 - 6·1) | 37 (36·6 - 37·4) | 11 (10·8 - 11·2) | 4 (3·9 - 4·1) |
| 2007 | 33 (32·8 - 33·2) | 11 (10·9 - 11·1) | 7 (6·9 - 7·1) | 26 (25·7 - 26·3) | 10 (9·8 - 10·2) | 10 (9·8 - 10·2) | 44 (43·6 - 44·4) | 13 (12·8 - 13·2) | 5 (4·9 - 5·1) |
| 2008 | 38 (37·7 - 38·3) | 13 (12·8 - 13·2) | 10 (9·9 - 10·1) | 30 (29·7 - 30·3) | 13 (12·8 - 13·2) | 13 (12·8 - 13·2) | 50 (49·6 - 50·4) | 15 (14·8 - 15·2) | 7 (6·8 - 7·2) |
| 2009 | 42 (41·7 - 42·3) | 16 (15·8 - 16·2) | 12 (11·9 - 12·1) | 34 (33·7 - 34·3) | 15 (14·8 - 15·2) | 16 (15·8 - 16·2) | 54 (53·6 - 54·4) | 18 (17·7 - 18·3) | 9 (8·8 - 9·2) |
| 2010 | 46 (45·7 - 46·3) | 18 (17·8 - 18·2) | 15 (14·8 - 15·2) | 38 (37·7 - 38·3) | 17 (16·8 - 17·2) | 20 (19·7 - 20·3) | 58 (57·5 - 58·5) | 20 (19·7 - 20·3) | 11 (10·8 - 11·2) |
| 2011 | 49 (48·7 - 49·3) | 20 (19·8 - 20·2) | 18 (17·8 - 18·2) | 41 (40·6 - 41·4) | 20 (19·8 - 20·2) | 23 (22·7 - 23·3) | 61 (60·5 - 61·5) | 22 (21·7 - 22·3) | 13 (12·8 - 13·2) |
| 2012 | 53 (52·7 - 53·3) | 22 (21·8 - 22·2) | 21 (20·8 - 21·2) | 44 (43·6 - 44·4) | 22 (21·7 - 22·3) | 27 (26·7 - 27·3) | 65 (64·5 - 65·5) | 24 (23·7 - 24·3) | 16 (15·8 - 16·2) |
| 2013 | 55 (54·7 - 55·3) | 24 (23·8 - 24·2) | 24 (23·8 - 24·2) | 47 (46·6 - 47·4) | 24 (23·7 - 24·3) | 31 (30·7 - 31·3) | 67 (66·5 - 67·5) | 26 (25·7 - 26·3) | 17 (16·8 - 17·2) |
| 2014 | 58 (57·7 - 58·3) | 26 (25·8 - 26·2) | 27 (26·8 - 27·2) | 51 (50·6 - 51·4) | 26 (25·7 - 26·3) | 35 (34·7 - 35·3) | 69 (68·5 - 69·5) | 27 (26·7 - 27·3) | 20 (19·8 - 20·2) |
| 2015 | 61 (60·7 - 61·3) | 28 (27·8 - 28·2) | 30 (29·8 - 30·2) | 54 (53·6 - 54·4) | 29 (28·7 - 29·3) | 39 (38·7 - 39·3) | 71 (70·5 - 71·5) | 29 (28·7 - 29·3) | 23 (22·7 - 23·3) |
| 2016 | 63 (62·7 - 63·3) | 30 (29·8 - 30·2) | 34 (33·8 - 34·2) | 57 (56·6 - 57·4) | 31 (30·7 - 31·3) | 42 (41·7 - 42·3) | 73 (72·5 - 73·5) | 30 (29·7 - 30·3) | 25 (24·7 - 25·3) |
| 2017 | 66 (65·7 - 66·3) | 32 (31·8 - 32·2) | 36 (35·8 - 36·2) | 61 (60·6 - 61·4) | 33 (32·7 - 33·3) | 46 (45·6 - 46·4) | 74 (73·5 - 74·5) | 32 (31·7 - 32·3) | 27 (26·7 - 27·3) |
| 2018 | 70 (69·7 - 70·3) | 34 (33·8 - 34·2) | 39 (38·8 - 39·2) | 66 (65·6 - 66·4) | 35 (34·7 - 35·3) | 50 (49·6 - 50·4) | 76 (75·5 - 76·5) | 34 (33·7 - 34·3) | 29 (28·7 - 29·3) |
| 2019 | 74 (73·7 - 74·3) | 36 (35·8 - 36·2) | 41 (40·8 - 41·2) | 72 (71·6 - 72·4) | 36 (35·7 - 36·3) | 52 (51·6 - 52·4) | 78 (77·5 - 78·5) | 36 (35·7 - 36·3) | 31 (30·7 - 31·3) |
| 2020 | 77 (76·7 - 77·3) | 37 (36·8 - 37·2) | 44 (43·8 - 44·2) | 77 (76·5 - 77·5) | 37 (36·7 - 37·3) | 55 (54·6 - 55·4) | 80 (79·5 - 80·5) | 37 (36·7 - 37·3) | 32 (31·7 - 32·3) |
| 2021 | 81 (80·7 - 81·3) | 39 (38·8 - 39·2) | 46 (45·8 - 46·2) | 83 (82·5 - 83·5) | 39 (38·7 - 39·3) | 59 (58·6 - 59·4) | 81 (80·5 - 81·5) | 39 (38·7 - 39·3) | 34 (33·7 - 34·3) |

Supplementary Table L. Age-standardised prevalence rates per 10,000 (95% confidence interval) of the number of people exposed to potentially cardiotoxic cancer medicines, excluding tamoxifen and aromatase inhibitors, and alive during each calendar year after N years (ie, 1, 5, and 10 years), exposed to N medicines (i.e., 1, 2, or 3 or more).

|  | One medicine | | Two medicines | | Three or more medicines | |
| --- | --- | --- | --- | --- | --- | --- |
| Year | Five-year prevalence | Ten-year prevalence | Five-year prevalence | Ten-year prevalence | Five-year prevalence | Ten-year prevalence |
| 2005 | - | - | - | - | - | - |
| 2006 | - | - | - | - | - | - |
| 2007 | - | - | - | - | - | - |
| 2008 | - | - | - | - | - | - |
| 2009 | 12 (11·7 - 12·3) | - | 4 (3·8 - 4·2) | - | 3 (2·8 - 3·2) | - |
| 2010 | 18 (17·7 - 18·3) | - | 6 (5·8 - 6·2) | - | 6 (5·8 - 6·2) | - |
| 2011 | 24 (23·7 - 24·3) | - | 7 (6·8 - 7·2) | - | 8 (7·8 - 8·2) | - |
| 2012 | 29 (28·7 - 29·3) | - | 9 (8·8 - 9·2) | - | 11 (10·8 - 11·2) | - |
| 2013 | 33 (32·7 - 33·3) | - | 10 (9·8 - 10·2) | - | 13 (12·8 - 13·2) | - |
| 2014 | 37 (36·7 - 37·3) | 6 (5·7 - 6·3) | 11 (10·8 - 11·2) | 3 (2·8 - 3·2) | 18 (17·8 - 18·2) | 3 (2·8 - 3·2) |
| 2015 | 41 (40·7 - 41·3) | 10 (9·7 - 10·3) | 13 (12·8 - 13·2) | 4 (3·8 - 4·2) | 18 (17·8 - 18·2) | 5 (4·8 - 5·2) |
| 2016 | 44 (43·6 - 44·4) | 13 (12·7 - 13·3) | 14 (13·8 - 14·2) | 5 (4·8 - 5·2) | 21 (20·8 - 21·2) | 7 (6·8 - 7·2) |
| 2017 | 47 (46·6 - 47·4) | 16 (15·7 - 16·3) | 15 (14·8 - 15·2) | 7 (6·8 - 7·2) | 24 (23·8 - 24·2) | 9 (8·8 - 9·2) |
| 2018 | 51 (50·6 - 51·4) | 19 (18·7 - 19·3) | 16 (15·8 - 16·2) | 8 (7·8 - 8·2) | 27 (26·7 - 27·3) | 12 (11·7 - 12·3) |
| 2019 | 54 (53·6 - 54·4) | 21 (20·7 - 21·3) | 18 (17·8 - 18·2) | 9 (8·8 - 9·2) | 30 (29·7 - 30·3) | 14 (13·7 - 14·3) |
| 2020 | 59 (58·6 - 59·4) | 24 (23·7 - 24·3) | 19 (18·8 - 19·2) | 10 (9·8 - 10·2) | 32 (31·7 - 32·3) | 16 (15·7 - 16·3) |
| 2021 | 64 (63·6 - 64·4) | 26 (25·7 - 26·3) | 21 (20·8 - 21·2) | 11 (10·8 - 11·2) | 35 (34·7 - 35·3) | 18 (17·7 - 18·3) |
