# Supplementary material for: Prevalence of Australians exposed to potentially cardiotoxic cancer medicines: a population-based cohort study
Source: Lancet Reg Health West Pac. 2023 Aug 2;39:100872. doi: 10.1016/j.lanwpc.2023.100872 (PMC10410507; doi:10.1016/j.lanwpc.2023.100872)
Supplement: Supplementary Figs. — A and B [file mmc2.docx]

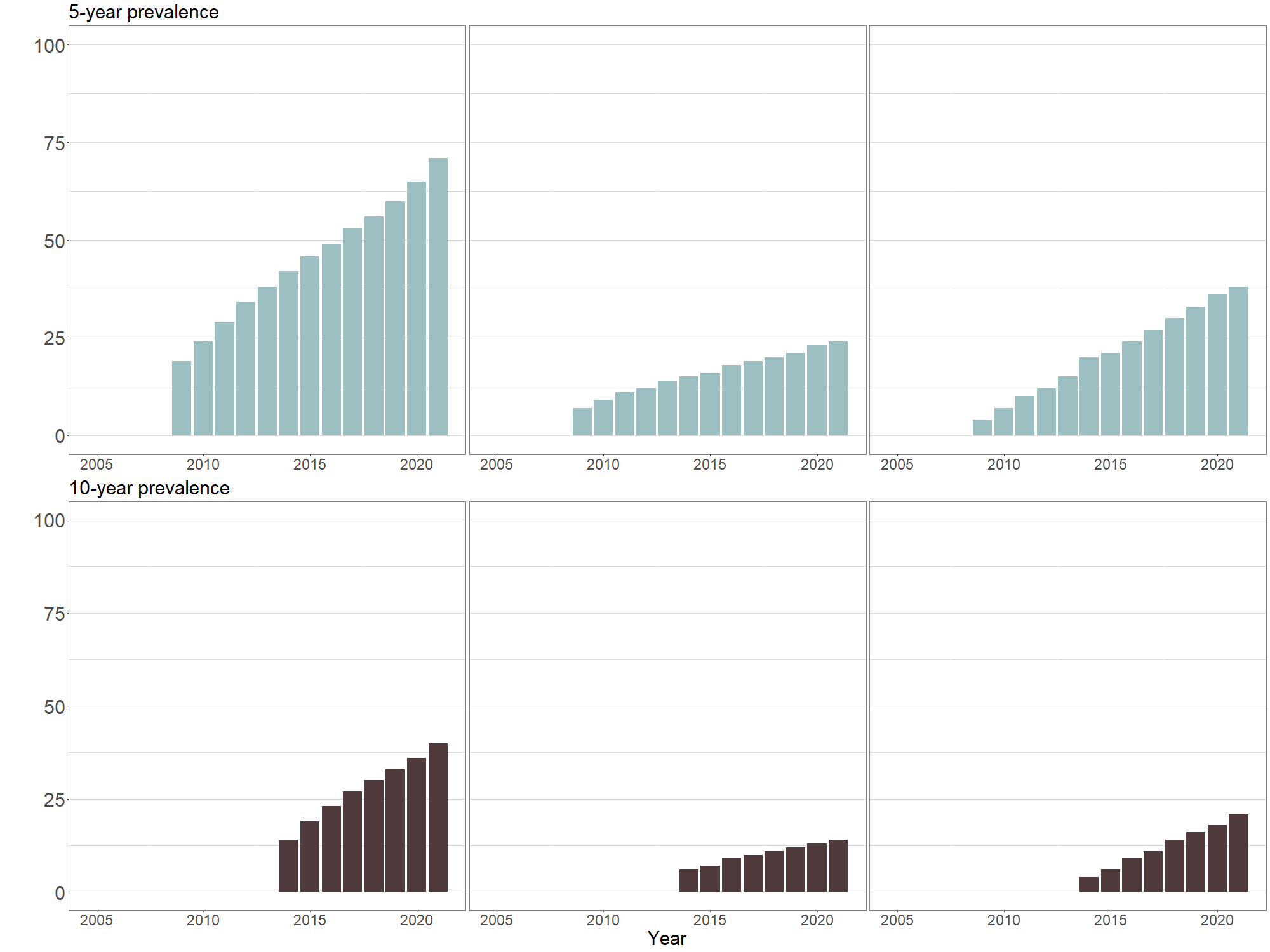


Supplementary Figure A. Age-standardised rates (per 10,000) of the number of people with previous exposure to potentially cardiotoxic pharmaceutical cancer medicines and alive during each year after five and 10 years, 2005 – 2021 (inclusive). Stratified by the number of potentially cardiotoxic medicines a patient was exposed to by that year.


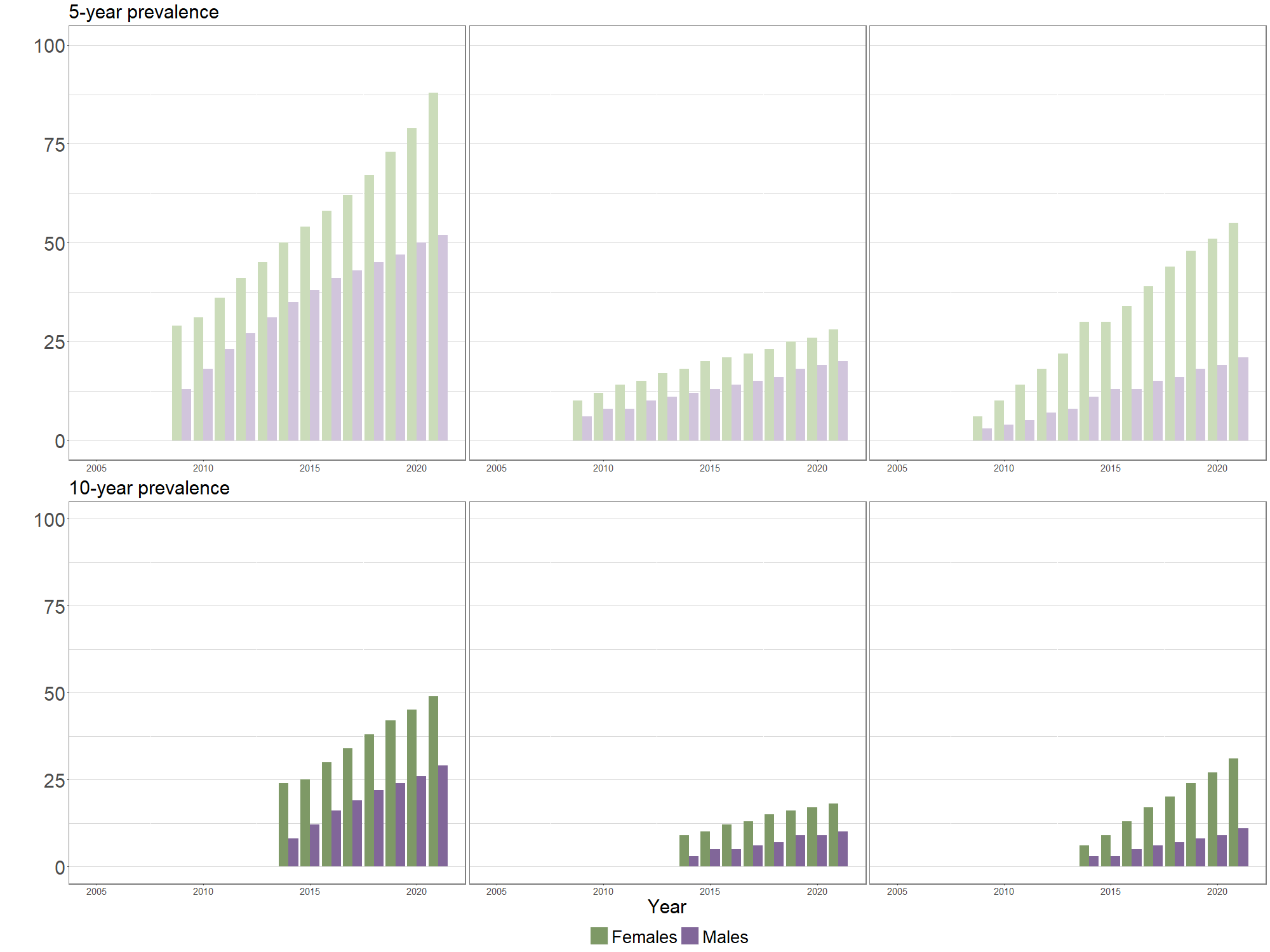


Supplementary Figure B. Age-standardised rates (per 10,000) of the number of people with previous exposure to potentially cardiotoxic pharmaceutical cancer medicines and alive during each year after five and 10 years, 2005 – 2021 (inclusive). Stratified by the number of potentially cardiotoxic medicines a patient was exposed to by that year and sex.
